# New High-Affinity Thrombin Aptamers for Advancing Coagulation Therapy: Balancing Thrombin Inhibition for Clot Prevention and Effective Bleeding Management with Antidote

**DOI:** 10.3390/cells12182230

**Published:** 2023-09-07

**Authors:** Mohamad Ammar Ayass, Natalya Griko, Victor Pashkov, Trivendra Tripathi, Jin Zhang, Ramya Ramankutty Nair, Tutku Okyay, Kevin Zhu, Lina Abi-Mosleh

**Affiliations:** Ayass Bioscience LLC, 8501 Wade Blvd, Building 9, Frisco, TX 75034, USA

**Keywords:** aptamer, thrombin, coagulation cascade, thrombosis, clot, VTE, PE

## Abstract

Thrombin is a key enzyme involved in blood clotting, and its dysregulation can lead to thrombotic diseases such as stroke, myocardial infarction, and deep vein thrombosis. Thrombin aptamers have the potential to be used as therapeutic agents to prevent or treat thrombotic diseases. Thrombin DNA aptamers developed in our laboratory exhibit high affinity and specificity to thrombin. In vitro assays have demonstrated their efficacy by significantly decreasing Factor II activity and increasing PT and APTT times in both plasma and whole blood. Aptamers AYA1809002 and AYA1809004, the two most potent aptamers, exhibit high affinity for their target, with affinity constants (Kd) of 10 nM and 13 nM, respectively. Furthermore, the in vitro activity of these aptamers displays dose-dependent behavior, highlighting their efficacy in a concentration-dependent manner. In vitro stability assessments reveal that the aptamers remain stable in plasma and whole blood for up to 24 h. This finding is crucial for their potential application in clinical settings. Importantly, the thrombin inhibitory activity of the aptamers can be reversed by employing reverse complement sequences, providing a mechanism to counteract their anticoagulant effects when necessary to avoid excessive bleeding. These thrombin aptamers have been determined to be safe, with no observed mutagenic or immunogenic effects. Overall, these findings highlight the promising characteristics of these newly developed thrombin DNA aptamers, emphasizing their potential for therapeutic applications in the field of anticoagulation therapy. Moreover, the inclusion of an antidote in the coagulation therapy regimen can improve patient safety, ensure greater therapeutic efficacy, and minimize risk during emergency situations.

## 1. Introduction

Thrombosis is a serious condition in which a blood clot forms inside a blood vessel (artery or vein). If thrombosis is untreated, the clot can slow, block, or travel in the blood flow, causing life-threatening emergencies such as thromboembolic stroke, heart attack, venous thromboembolism (VTE), or pulmonary embolism (PE). It is estimated that 900,000 Americans are affected by VTE each year and that 100,000 Americans die from PE [1]. In recent years, the treatment of thrombosis has been gradually changing from conventional anticoagulants (heparin, vitamin K antagonists) to direct oral anticoagulants (DOACs) (oral direct thrombin inhibitors (DTIs), oral factor Xa inhibitors). In comparison to conventional anticoagulant therapies, DOACs have the advantages of oral administration, no need for frequent monitoring and dose adjustment, and fewer medication and food interactions [2].

Among the activated coagulation proteinases, thrombin is a unique coagulation protein that plays a central orchestrating role in procoagulation, anticoagulation, and platelet activation [3]. In procoagulation, thrombin converts fibrinogen into insoluble fibrin clots while activating platelets and factors XIII, V, VIII, and XI. In anticoagulation, thrombin plays a regulatory and control role in the coagulation cascade by activating protein C, which together with its cofactor protein S degrades FVIIIa and FVa. The multiple roles of thrombin are mostly attributed to its highly dynamic three-dimensional molecular structure [3,4,5]. Thrombin has a catalytic site and two anion-binding active sites, exosite I and exosite II. Exosite I is the active site of thrombin, and is responsible for interacting with various molecules, including fibrinogen, fibrin, heparin cofactor II, and protease-activated receptor [6]. Exosite II is the specific site responsible for the activation of factors V factor VIII, as well as for heparin binding.

Dabigatran etexilate (Pradaxa) is an FDA-approved direct and reversible thrombin inhibitor. It is an orally administered anticoagulant specifically targeting thrombin, and has been approved for various indications, including prevention of systemic embolism in non-valvular atrial fibrillation, prevention of recurrent venous thromboembolism (VTE), and prevention of stroke. Dabigatran is an active form of dabigatran etexilate that competitively and selectively binds thrombin, preventing the conversion of soluble fibrinogen to insoluble fibrin and inhibiting thrombin-induced platelet aggregation [7]. It is a fast-acting drug and can be rapidly reversed with idarucizumab, a monoclonal antibody that binds to dabigatran and neutralizes its anticoagulant properties. In the past decade of clinical application, research has found that dabigatran shares the same side effects, drug interactions, and limitations as DOACs. Taking dabigatran may cause stomach discomfort, bloody stools, pain or a burning sensation in the throat, etc. [8]. Moreover, dabigatran should be avoided/is contraindicated in patients with creatine clearance (CrCl < 30 mL/min), pregnancy, mechanical heart valves, and/or a history of serious dabigatran hypersensitivity [9]. Furthermore, due to significant drug interactions there are over 150 medications that are not recommended for use with dabigatran (e.g., Abciximab, Capmatinib, Dalteparin, etc.). Finally, taking dabigatran increases the risks of bleeding, which could potentially be fatal in certain cases. Recent studies have found that there are no differences between DOACs and conventional anticoagulants in terms of their safety and long-term efficacy in preventing recurrent PE/VTE, mortality, and major bleeding [10,11,12]. Therefore, development of effective and safe DOACs remains an ongoing challenge in the context of reducing both short-term and long-term negative outcomes associated with thrombosis.

Aptamers are RNA and DNA oligonucleotides that bind to specific target molecules with high affinity and specificity, in which they are similar to antibodies; however, they have many advantages over antibodies, such as long shelf life, ease of modification, cost-effectiveness, and lower immunogenicity. In the coagulation cascade, aptamers have been developed to interact with multiple coagulation factors and cofactors, offering antidote-mediated controllability that safeguards the intrinsic coagulation capacity from exhaustion [13,14]. As a result, aptamers have recently drawn a great deal of attention for their potential to regulate the activity of thrombin.

The first anti-thrombin DNA aptamer (HD1, 5${}^{\prime }$-GGTTGGTGTGGTTGG-3${}^{\prime }$) was synthesized by Dr. Toole in 1992; it is a 15-mer aptamer that forms a stable G-quadruplex binding to exosite I on prothrombin and thrombin [15,16]. HD1 demonstrates a comprehensive inhibitory effect by targeting multiple pathways, including fibrinogen and FV cleavage, prothrombinase inhibition, and blocking platelet PARI interaction with exosite I. This multifaceted action results in the effective suppression of thrombin-mediated platelet activation and aggregation [17]. In vivo studies have shown that HD1 inhibits >80% of clot-bound thrombin, as compared to 35% with heparin [18,19,20]. Due to its short half-life and rapid clearance, HD1 needs to be administered by infusion in large amounts, which poses a challenge in the dosing and monitoring of the drug. For these reasons, HD1 was ruled out for phase I clinical trials. Additional anti-thrombin aptamers that bind to exosite I of thrombin were identified later, such as NU172 [21], RA36 [22], R9D-14T [23], and RE31 [24]. Among these, NU172 is the only aptamer that entered phase II clinical trials (NCT00808964) [25]; at present, there is a lack of available information on its current status.

HD22 (29-mer, 5${}^{\prime }$-AGTCCGTGGTAGGGCAGGTTGGGGTGACT-3${}^{\prime }$) is another well-known aptamer. This aptamer recognizes exosite II of thrombin, a crucial region for activation of factors V and VIII, as well as for the mediation of heparin binding. As a result, HD22 inhibits the activation of factors V and VIII rather than that of fibrinogen [26]. Toggle-25t, an RNA aptamer, is another aptamer that binds to exosite II of thrombin [27].

To enhance the inhibitory potency of aptamers, studies have targeted multiple sites of thrombin for inhibition. As a result, several anti-thrombin bivalent aptamers have been designed by chemically linking an aptamer that binds to exosite I with another aptamer that targets exosite II. Examples of such aptamers include HD1-22 [28], a bivalent aptamer that connects HD1 and HD22(Kd = 0.65 nM), as well as RNV220 and RNV220-T, both bivalent aptamers that connect HD1 and HD22 by TEG or poly-dT linkage [29]. Recently, another bivalent aptamer linking M08s-1 and TBA29 was developed for heparin-induced thrombocytopenia, showing significantly stronger anticoagulant activity than NU172 [30].

While numerous anti-thrombin aptamers have been designed and developed, no anti-thrombin aptamer has reached advanced stages in clinical trials or received approved from the FDA. One major challenge in their clinical translation pertains to their susceptibility to nuclease degradation and compromised pharmacokinetic properties, requiring higher dosing regiments. Consequently, the development of new thrombin aptamers remains crucial as a means to broaden the range of therapeutic options, enhance treatment effectiveness, overcome existing limitations, and propel the field towards more personalized and effective approaches in the management of thrombotic disorders.

In this study, we employed systematic evolution of ligands by exponential enrichment (SELEX) to select aptamers that bind to the active site of thrombin. Several anti-thrombin aptamers with binding high affinity were successfully generated. The two most potent aptamers, AYA1809002 and AYA1809004, exhibit high affinity for their target, with affinity constants (Kd) of 10 nM and 13 nM, respectively. Furthermore, they demonstrate in vitro assay activity by effectively decreasing Factor II activity and increasing both prothrombin time (PT) and activated partial thromboplastin time (APTT) in plasma and whole blood in a dose-dependent manner. Crucially, the thrombin inhibitory activity of these aptamers can be effectively reversed through the use of reverse complement sequences. This feature provides a valuable mechanism to counteract their anticoagulant effects, enabling timely and precise intervention in emergency scenarios. Our results show that these two aptamers are stable in whole blood at room temperature and 37 °C for at least 24 h. To enhance their stability in the bloodstream, both aptamers were chemically modified through crosslinking with TAG, PEG, or cholesterol moieties. These modified aptamers successfully demonstrated in vitro activity, indicating that they retain their functionality. Additionally, both of these thrombin aptamers were determined to be safe, without any observed immunogenic or mutagenic effects. Our findings suggest that AYA1809002 and AYA1809004 are effective and safe anti-thrombin candidate aptamers that can potentially be used to treat thrombosis in the future.

## 2. Materials and Methods

### 2.1. Immobilization of Thrombin Protein on Magnetic Beads

The immobilization of human alpha-thrombin native protein (ThermoFisher Scientific, cat# RP-43100, Waltham, MA, USA) on PierceTM NHS-Activated Magnetic Beads (ThermoFisher Scientific, cat# 88827, Waltham, MA, USA) was performed as described by standard protocol, as follows. One mg of thrombin (MW 36.7 kDa) was dissolved in coupling buffer (0.1 M NaHCO${}_{3}$, pH 8.3 containing 0.15 M NaCl) to a concentration of 0.7 mg/mL and dialyzed against 500 mL coupling buffer overnight. One ml of magnetic beads (10 mg of beads) was placed on the magnetic stand, the supernatant was discarded, and the beads were resuspended in 3 mL of ice-cold 1 mM hydrochloric acid, vortexed for 15 s, and placed on the magnetic stand to collect the beads; the supernatant was then discarded. Coupling solution containing thrombin was added to the functionalized beads and the mixture was rotated for 2 h at room temperature. The excess of ligand was washed out with coupling buffer followed by 0.1 M glycine buffer, pH 2.5. Remaining active groups were blocked using 3 M ethanolamine, pH 9.0. After 2 h, the incubation beads were washed and resuspended in selection buffer (20 mM Tris pH 7.5, 150 mM NaCl). Binding of thrombin was quantitated and confirmed using Pierce BCA protein Assay kit (ThermoFisher Scientific, cat# 23227, Waltham, MA, USA). The final concentration was estimated as 36 µg of protein per mg beads, or 1 nmol of protein per mg of beads. The same procedure was performed with the beads without thrombin protein in coupling buffer for negative selection.

### 2.2. SELEX

The systematic evolution of ligands by exponential enrichment (SELEX) procedure was performed as previously described in [31]. A synthetic single stranded DNA library consisted of a random sequence of 40 nucleotides flanked by two primers binding sequences 5${}^{\prime }$ TAGGGAAGAGAAGGACATATGAT(N40)TTGACTAGTACATGACCACTTGA 3${}^{\prime }$ (TriLink biotechnologies, cat# O-32140, 9955 Mesa Rim Road, San Diego, CA 92121, USA), and 5${}^{\prime }$-Biotin Reverse primer was used for single strand separation. Throughout the subsequent SELEX rounds, the initial ssDNA library (10 nmol) was dissolved in 100 µL of DNase and RNase free water plus 100 µL of selection buffer (20 mM Tris pH 7.5, 150 mM NaCl). The diluted library was denatured using Hybex incubator at 95 °C for 10 min and cooled slowly on ice for 10 min then at room temperature for 30 min to allow the formation of stable secondary structures. Selection buffer was added up to 1 mL total volume of the library. This library was incubated with 300 µL (3 mg) of the magnetic beads (no thrombin) equilibrated with selection buffer for 2 h at room temperature (negative selection). The beads were collected using the magnetic stand and the supernatant was applied to 1 mg of the thrombin beads equilibrated with selection buffer. After 2 h incubation at room temperature and washing in the selection buffer, the bound ssDNA pool was eluted under alkaline conditions (200 µL of 0.15 M NaOH). The elution was added into a tube containing 200 µL of 0.15 M of acetic acid. Overnight ethanol precipitation was performed at −20 °C and the pellet was collected by centrifugation, washed with 70% ethanol, and dissolved in 15 µL of water; 2 µL of ssDNA was used for PCR amplification, and the rest was stored at −20 °C. The selected ssDNA was PCR amplified using PureTaq ready-to-go PCR Beads (GE Healthcare, cat # 27-9557-01, Amersham Place, Little Chalfont, Buckinghamshire HP7 9NA, UK) in a volume of 25 µL with 2 µL of 10 µM Forward primer, 2 µL of 10 µM 5${}^{\prime }$-Biotin Reverse primer, 2 µL ssDNA, and 19 µL H${}_{2}$O (25 cycles of 30 s at 95 °C, 30 s at 50 °C, 30 s at 72 °C, and finally 5 min at 72 °C). The PCR product was converted to single-stranded DNA by using Dynabeads(TM) M-280 Streptavidin magnetic beads (ThermoFisher Scientific, cat# 11206D, Waltham, MA, USA, standard protocol). The resulting product formed a new enriched library pool that was used for subsequent rounds of SELEX. The process was repeated for nine cycles with increasing selection stringency by increasing the stringency of the washing buffer to 0.5 M NaCl and increasing the wash time. To enhance the specificity of the selected oligonucleotides, counter-selection was performed after the fourth round with thrombin-depleted serum (PrecisionBiologic, cat # FPD02-10, Dartmouth, NS B3B 0A9, Canada) proteins immobilized on magnetic beads. The second counter selection was performed after the sixth round to enrich the library with aptamers that bind to the active site of thrombin. We employed dabigatran, a drug that directly binds to the active site of thrombin, effectively blocking the binding of aptamers to that specific site. Dabigatran (5 nmol) was applied to thrombin beads and blocked thrombin active sites. ssDNA from the sixth round of SELEX was loaded onto the beads and the flow throw was collected. Collected libraries that did not bind to the dabigatran–thrombin complex were subsequently subjected to thrombin bead application, which allowed us to continue the positive selection process, specifically targeting aptamers that exhibited robust binding to thrombin. The SELEX process then proceeded for three more rounds of traditional selection.

### 2.3. Next-Generation Sequencing of SELEX Samples

The single-stranded DNA (ssDNA) obtained after each round of SELEX underwent library preparation for Illumina sequencing using the TruSeq ChIP sample preparation protocol. A total of nine DNA libraries comprised of paired-end indexed sequences were created by pooling the samples. The Illumina TruSeq ChIP sample preparation kit reagents were utilized for this process to facilitate cluster generation and subsequent DNA sequencing. The initial DNA input (50 µL of 200 pg/µL) underwent processes to blunt-end and phosphorylate the molecules. Additionally, a single “A” nucleotide was attached to the 3${}^{\prime }$ ends of the fragments, preparing them for ligation to adapters featuring a single-base “T” overhang. These adapter sequences were introduced to the DNA ends to generate either indexed single-read or paired-end sequencing libraries. Following ligation, the resulting products were purified and accurately size-selected using agarose gel electrophoresis. The DNA fragments of the desired size were isolated and purified once more. Subsequently, a PCR amplification step was performed to enrich for fragments possessing adapters on both ends. The final product underwent quantification prior to the initiation of cluster generation.

### 2.4. ELISA-Based Binding Assay of Biotinylated Thrombin Aptamers to Thrombin Protein

Native human alpha-thrombin protein (ThermoFisher Scientific, cat# RP-43100, Waltham, MA, USA) was added to each well of a 96-well plate (Nunc MaxiSorp flat-bottom 96-well plates, ThermoFisher Scientific, cat# 44-2404-21, Waltham, MA, USA) at a concentration of 250 nM in Tris-buffered saline with a volume of 100 µL per well. The plate was then incubated at 4 °C for 16 h to allow the wells to become coated with thrombin. After removing the thrombin solution, the wells were washed and blocked using blocking buffer (20 mM Tris, 150 mM NaCl pH 7.5, 2% BSA, 0.1% Tween 20 and 100 µg/mL of sheared salmon sperm DNA) for a 1 h duration. Next, biotinylated aptamers at the indicated concentrations in a volume of 100 µL were added to the wells in an incubation buffer (20 mM Tris, 150 mM NaCl pH 7.5, 0.1% BSA, 0.1% Tween 20 and 100 µg/mL of sheared salmon sperm DNA) and incubated for 1 h at room temperature. In the competition binding assay, unlabeled aptamers were added along with the biotinylated aptamers, with the unlabeled aptamers being present in a 100-fold excess compared to the biotinylated aptamers. Following three washing steps with wash buffer (20 mM Tris, 150 mM NaCl pH 7.5, 0.1% BSA and 0.1% Tween 20), streptavidin-HRP (ThermoFisher Scientific, cat# 21130, Waltham, MA, USA) was added at a dilution of 1:5000 in the incubation buffer. The plate was washed again three times to remove any unbound reagents. The bound biotinylated aptamers were then detected using 3,3${}^{\prime }$,5,5${}^{\prime }$-Tetramethylbenzidine (TMB) substrate (ThermoFisher Scientific, cat# 34021, Waltham, MA, USA) following the manufacturer’s instructions.

### 2.5. Secondary Structure Prediction

The RNAfold webserver was used to predict the secondary structure of the aptamers. This server is available online and can be used for RNA and ssDNA prediction. Minimum Free Energy (MFE) secondary structure prediction aims to find the structure with the lowest free energy. It predicts the structure by minimizing the thermodynamic free energy of the nucleic acid molecule.

### 2.6. In Vitro Activity Assay

Sterile human blood in sodium citrate was purchased from the Rockland Immunochemicals, Inc (cat # R214-0050, Pottstown, PA 19464, USA). Plasma was prepared by centrifugation for 10 min at 2000× *g*. In vitro activity assays were performed on ACLTOP analyzer manufactured by Instrumentation Laboratory, which is a type of coagulation analyzer commonly used in clinical laboratories to measure clotting times and assess coagulation parameters. The ACLTOP analyzer is designed to perform a wide range of coagulation tests, including Factor II activity, prothrombin time (PT), activated partial thromboplastin time (APTT), fibrinogen, and other related assays. ACLTOP analyzer uses HemosIL reagents developed by Instrumentation Laboratory company (Bedford, MA, USA) to perform coagulation tests.

#### 2.6.1. Factor II Activity Assay

For screening in vitro activity of the aptamers, human citrated plasma (0.5 mL) was incubated in the absence or presence of 2 µM thrombin aptamers or dabigatran for 2 h. Factor II activity was measured using human plasma immunodepleted of Factor II for the quantitative determination of Factor II activity in citrated plasma based on the prothrombin time (PT) assay, for which an ACLTOP coagulation analyzer manufactured by Instrumentation Laboratory (Bedford, MA, USA) was used. To evaluate the dose-dependent activity of the aptamers in whole blood, human blood samples were collected in citrate-treated tubes. Subsequently, the blood samples were incubated at room temperature for 2 h in both the absence and presence of various concentrations of thrombin aptamers. To measure the Factor II activity using the ACLTOP coagulation analyzer, plasma was separated from the cells by centrifugation at 1500× *g* for 10 min at 4 °C. For the dose-dependent study, concentrations of 1 µM, 2 µM, 3 µM, and 4 µM of AYA1809002 and AYA1809004 were utilized.

#### 2.6.2. Prothrombin Time Assay

Human citrated plasma (0.5 mL) was incubated in the absence or presence of 2 µM thrombin aptamers or dabigatran for 2 h. PT time was measured using RecombiPlasTin 2G for quantitative determination in human citrated plasma of PT and Fibrinogen on an ACLTOP coagulation analyzer manufactured by Instrumentation Laboratory (Bedford, MA, USA). To assess the activity of the aptamers in a dose-dependent manner in whole blood, human blood samples were collected in citrate-treated tubes. The blood samples were then incubated at room temperature for 2 h in the absence or presence of varying concentrations of the thrombin aptamers. To measure the Prothrombin Time (PT) on the ACLTOP coagulation analyzer, plasma was obtained by centrifuging the samples at 1500× *g* for 10 min at 4 °C. For the dose dependence study, concentrations of 1 µM, 2 µM, 3 µM, and 4 µM of AYA1809002 and AYA1809004 were utilized.

#### 2.6.3. Activated Partial Thromboplastin Time (APTT) Assay

Human citrated plasma (0.5 mL) was incubated in the absence or presence of 2 µM thrombin aptamers or dabigatran for 2 h. APTT time was measured using the synthetic phospholipid reagent SynthASIil for quantitative determination in human citrated plasma on an ACLTOP coagulation analyzer manufactured by Instrumentation Laboratory (Bedford, MA, USA).

### 2.7. Direct Thrombin Activity Inhibition

A Thrombin Inhibitor Screening Assay Kit (fluorometric) (Abcam, cat #ab197007, Waltham, Boston, MA, USA) was employed to test the direct binding ability and inhibitory potential of the AYA1809002 and AYA1809004 aptamers against thrombin. The procedure was carried out following the standard protocol provided with the kit. In brief, aptamers at different concentrations, dabigatran at 2 µM, and controls were added to their respective wells on the plate (ThermoFisher Scientific, cat # 3915, Assay Plate, Black, Flat Bottom, Waltham, MA, USA). Subsequently, a thrombin enzyme mixture was added to each sample and control well. Following an incubation period of 20 min, a substrate mixture was introduced into each sample and control well. Fluorescence measurements were taken using a SYNERGY/HTX multi-mode reader (BioTek, Agilent Technologies, Santa Clara, CA 95051, USA) with the excitation/emission wavelengths set at 360/460 nm. The measurements were recorded for 45 min at a temperature of 37 °C while ensuring protection from light.

### 2.8. Stability Study of Selected Aptamers in Whole Blood at Room Temperature and 37 °C

To evaluate the stability of the selected aptamers (AYA1809002, AYA1809004) and compare them to dabigatran, citrated blood collected from a donor was incubated at room temperature in the absence or presence of 1 µM of AYA1809002, AYA1809004, or dabigatran for 0.5, 2, 4, 6, and 24 h. At each time point, blood samples from both the treated and untreated groups were collected and plasma was obtained by centrifugation. The collected plasma samples were then frozen for further analysis. Factor II activity was measured using human plasma immunodepleted of Factor II for quantitative determination of Factor II activity in citrated plasma based on the prothrombin time (PT) assay, for which an ACLTOP coagulation analyzer manufactured by Instrumentation Laboratory was used. PT time was measured using RecombiPlasTin 2G for quantitative determination in human citrated plasma of PT and Fibrinogen on an ACLTOP coagulation analyzer manufactured by Instrumentation Laboratory. The stability experiments at 37 °C were conducted as follows: citrated blood was collected from a donor and incubated with or without 1 µM AYA1809002 or AYA1809004 at 37 °C. At each time point, blood samples from both the treated and untreated groups were collected and plasma was obtained by centrifugation. Factor II activity, PT time, and APTT time were measured for each sample. To assess the relative activity, the measured activity of the treated whole blood sample was divided by the activity of the corresponding untreated sample collected at the same time point.

To provide additional evidence of the aptamers’ stability in an in vitro setting, we conducted the same stability assay using whole blood at 37 °C. At various time intervals, we utilized biotinylated reverse complement sequences along with streptavidin-coated magnetic beads to selectively isolate the aptamers from the blood samples. Following elution from the magnetic beads, the aptamers underwent analysis using a Fragment Analyzer system (Agilent Technologies, Santa Clara, CA 95051, USA) to assess their integrity.

### 2.9. Restoration of Factor II Activity and PT Time with a Reverse Complement to AYA1809002 in Whole Blood

Citrated blood collected from a donor was incubated in the absence or presence of 1 µM AYA1809002 at room temperature. After 2 h incubation, the indicated concentration of the reverse complement strand was added to the citrated blood sample. After an additional incubation time of 2 h, plasma was obtained through centrifugation. Factor II activity was measured using human plasma immunodepleted of Factor II for the quantitative determination of Factor II activity in citrated plasma based on the prothrombin time (PT) assay, for which an ACLTOP coagulation analyzer manufactured by Instrumentation Laboratory (Bedford, MA, USA) was used. The PT time was measured using RecombiPlasTin 2G for quantitative determination in human citrated plasma of PT and Fibrinogen on an ACLTOP coagulation analyzer manufactured by Instrumentation Laboratory (Bedford, MA, USA). As a control, 1 µM of dabigatran was incubated with the citrated blood.

### 2.10. Clot-Bound Thrombin

For the inhibition study of clot-bound thrombin, we utilized the method previously described in [32,33] with a few modifications. In brief, blood samples were collected from healthy donors using citrate-treated tubes and centrifuged at 200× *g* for 15 min at room temperature. The resulting supernatant consisting of platelet-rich plasma (PRP) was collected and aliquots of 500 µL were transferred to microfuge tubes containing 50 µL of 300 mM CaCl${}_{2}$. The mixture was then incubated for 2 h at 37 °C in an incubator without agitation. The formed clots were washed ten times over the course of 16 h at room temperature; each time, 1 mL aliquots of TBS buffer (50 mM Tris-HCl, 100 mM NaCl, pH 7.4) were added to the clots with mixing. Subsequently, the washed clots were transferred to the assay plate (ThermoFisher Scientific, cat # 3915, Waltham, MA, USA). Different concentrations of selected aptamers and 2 µM of dabigatran in 200 µL of TBS buffer were added to their respective wells containing the washed clots. After a 20 min incubation period with agitation, a thrombin substrate (Thrombin substrate III, Fluorogenic, Sigma-Aldrich, cat # 605211, St. Louis, MO, USA) was added to each sample at a final concentration of 200 µM. Fluorescence measurements were performed using a SYNERGY/HTX multi-mode reader (BioTek, Agilent Technologies, Santa Clara, CA 95051, USA) with the excitation/emission wavelengths set at 360/460 nm. The measurements were recorded for a total duration of 90 min at a temperature of 37 °C.

### 2.11. Effect of Modified AYA1809002 and AYA1809004 Aptamers on FACTOR II Activity, PT, and APTT in Human Citrated Plasma and Reversal Potential Using Respective Reverse Complement for Each Aptamer

Modified aptamers chemically crosslinked with TAG, PEG, and cholesterol entities at the 3${}^{\prime }$ end, were purchased from IDT (Integrated DNA Technologies, Coralville, IA, USA). The impact of modified AYA1809002 and AYA1809004 aptamers on Factor II activity, PT, and APTT was evaluated using the same protocol as was used for the unmodified aptamers. Additionally, the potential for reversal was investigated using the respective reverse complement for each aptamer.

### 2.12. Exploring the Immunogenic Reaction of AYA1809002 and AYA1809004 on Human Peripheral Blood Mononuclear Cells (hPBMCs)

Human PBMCs were isolated from buffy coats provided by Carter BloodCare(Bedford, TX, USA). Isolation was carried out through density-gradient centrifugation utilizing Ficoll-Paque Plus from GE Healthcare. The isolated human PBMCs suspended in cRPMI media were subjected to stimulation with or without the AYA1809002 and AYA1809004 aptamers and a control aptamer. Various concentrations (1, 5, and 10 µM) of these aptamers were used, along with positive control stimuli such as LPS (200 ng/mL), ODN 1826 (20 µM, Invitrogen, Waltham, MA, USA), or a combination of LPS (100 ng/mL) and ODN 1826 (10 µM). Following incubation periods of 24 and 72 h, the cell supernatant was collected via centrifugation at 200 g for 3 min. Cytokine levels were evaluated using the LEGENDplex Human Inflammation Panel 1 from BioLegend (cat# 740808, San Diego, CA, USA) following the manufacturer’s protocol, soluble analytes were acquired using a Navios EX flow cytometer (Beckman Coulter Inc., Pasadena, CA, USA), and subsequent analysis was performed using BioLegend’s LEGENDplex${}^{\mathrm{TM}}$ system (San Diego, CA, USA).

### 2.13. Ames Test

The mutagenic potential of AYA1809002 and AYA1809004 was evaluated using the Ames test with the Xenometrix Ames MPF PENTA I kit, including S9 and Positive Controls (Aniara Diagnostica, code: AC01-512-S2-P; manufacturer’s part number: C01-512-S2-P, West Chester Township, OH). The assay procedure followed the protocol outlined in our previous publication [34].

### 2.14. Statistical Analysis

Statistical significance was assessed using GraphPad Prism version 10.0.0 (131), 2023 (GraphPad Software, Boston, MA, USA) employing nonparametric methods such as the Mann–Whitney test or unpaired Student’s *t*-test for comparisons between two groups. Data are presented as mean ± standard deviation (SD). A *p*-value of ≤0.05 was deemed statistically significant, and is indicated by an asterisk (*), while a *p*-value of ≤0.01 is denoted by a double asterisk (**).

## 3. Results

### 3.1. Developing High-Affinity Neutralizing DNA Aptamers against Human Thrombin Protein

To identify single-stranded DNA aptamers capable of binding to human thrombin protein, we employed the SELEX procedure with certain adaptations, as outlined in [35]. This process involves immobilizing human alpha-thrombin protein onto Pierce^TM^ NHS-Activated Magnetic beads following established protocols. It is worth noting that the immobilization process of thrombin on NHS beads involves a random attachment in which lysine (Lys) residues can potentially bind to NHS groups on the beads. In light of the steric challenges posed by the structure of activated thrombin, specifically the deep groove of its active site, utilizing lysine residues located within or near the active site for binding to the beads might be intricate. However, even if such binding occurs, it is important to note that other molecules of thrombin with accessible binding sites remain available for subsequent binding of aptamers [36].

**Figure 1:**
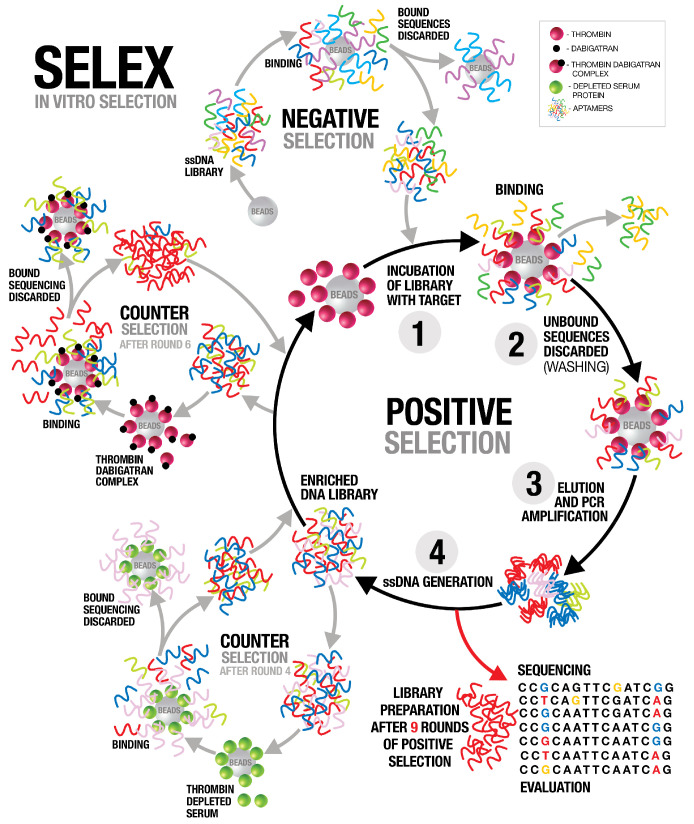
Illustration of the SELEX procedure used to develop the human alpha-thrombin-specific aptamers.A library of single-stranded DNA oligonucleotides (10${}^{15}$ different unique sequences) was used. Each unique sequence contains random bases (40 nt) flanked by two conserved primer binding sites, which are used for PCR amplification. Negative selection was performed using bare magnetic beads (no thrombin) to remove nonspecific interactions between DNA oligonucleotides and magnetic beads. Human alpha-thrombin native protein was immobilized on NHS-Activated Magnetic Beads. In the first positive selection step, the library was incubated with human thrombin protein immobilized on NHS-activated magnetic beads (**1**), the unbound sequences were separated from the bound ones (**2**), and target-bound sequences were eluted from target molecules and amplified by PCR using biotinylated reverse primer (**3**). The PCR product was pulled down using streptavidin beads, and the specific single stranded DNA was separated using sodium hydroxide buffer and utilized in the next round of selection (**4**). The process was repeated for nine rounds with increasing selection stringency by increasing the stringency of the washing buffer to 0.5 M NaCl and increasing the washing time. To enhance the specificity of the selected oligonucleotides, counter-selection was performed after the fourth round using thrombin depleted serum proteins immobilized on magnetic beads. A second counter-selection was performed after the sixth round to enrich the library with aptamers for the active site of thrombin. We used dabigatran, a drug that binds directly to active site of thrombin. Dabigatran (5 nmol) was applied to thrombin beads to block the thrombin active site. ssDNA from the sixth SELEX round were loaded onto the beads and the flow-through was collected, followed by three further rounds of traditional selection.

The selection of specific aptamers was carried out using a library of single-stranded DNA oligonucleotides encompassing 10${}^{15}$ distinct random sequences. These sequences were flanked by two 23-base primer sequences to facilitate selection. In order to enable the unique conformational folding of ssDNA, for the 10 nmol ssDNA library a heating step at 95 °C was followed by cooling on ice. The introduction of bare magnetic beads to the activated ssDNA library was employed to prevent the enrichment of aptamers that solely recognize the beads (negative selection). The SELEX procedure utilized thrombin-coated magnetic beads as the target (Figure 1). A gradual increase in selection stringency was achieved by raising the wash buffer’s NaCl concentration to 0.5 M and increasing the number of wash cycles. After four conventional selection rounds, counter-selection was carried out using magnetic beads immobilized with thrombin-depleted serum proteins. Following the fourth selection round, the eluted ssDNA was subjected to an incubation with thrombin-depleted serum proteins immobilized on magnetic beads. Flow-through was collected for the subsequent selection round after a one-hour incubation. After six rounds of conventional selection, dabigatran was introduced to the thrombin protein immobilized on the beads to block the active site. This complex was employed in order to select those aptamers specifically binding to the active site of thrombin protein (Figure 1). The eluted ssDNA from the sixth selection round was then incubated with the thrombin–dabigatran complex immobilized on the beads. The flow-through representing aptamers that exclusively bind to the active site of thrombin concealed by dabigatran was collected, then selection proceeded for an additional three rounds. The single-stranded DNA pools eluted from each selection round were chosen for high-throughput sequencing using the Illumina MiSeq platform. Sequence analysis was conducted based on the FASTAptamer method to identify sequences exhibiting enrichment across the various selection pools [37]. The top nine most enriched sequences after nine rounds of selection are listed in Table 1.

### 3.2. Aptamers Binding to Thrombin

**Figure 2:**
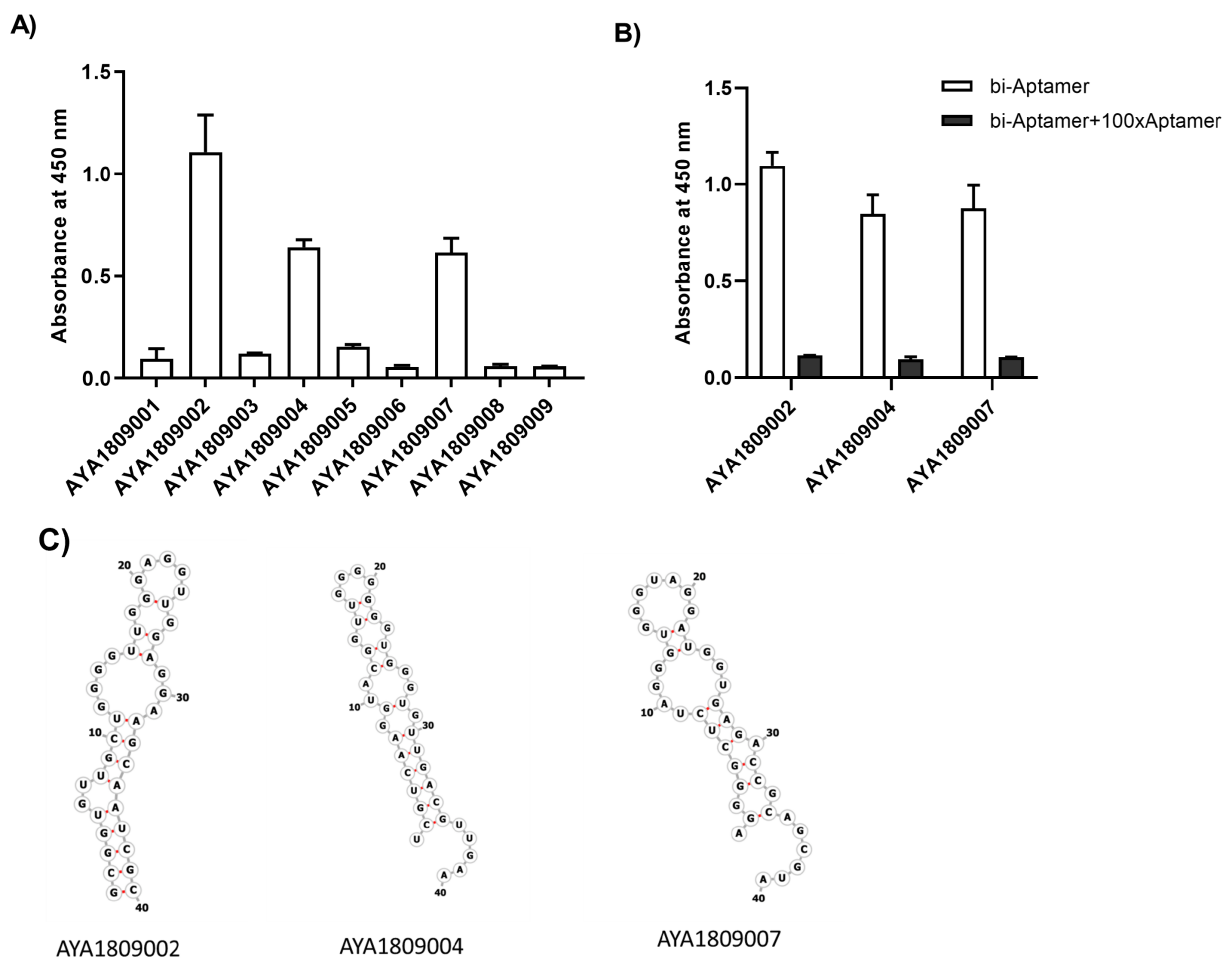
Binding of aptamers to thrombin. (**A**) Binding of the top nine enriched aptamer sequences to thrombin protein: thrombin protein was immobilized on a 96-well plate (see materials and method); the top nine enriched aptamers from the Next Generation Sequencing results were synthesized with a 3${}^{\prime }$ biotin group and incubated with the immobilized thrombin in duplicates. Absorbance was measured after incubation with streptavidin horseradish peroxidase bound to the biotinylated aptamer in the presence of the TMB substrate. Each bar shows the average of duplicate measurements. (**B**) Competition assay for aptamer binding to thrombin with an excess of non-biotin labelled aptamers. The specific binding of four aptamers that displayed binding to thrombin was tested in the presence of a 100-fold excess of the corresponding nonlabeled aptamer. All three aptamers show specific binding to thrombin that can be competed out in the presence of nonlabeled aptamer. Each bar shows the average of duplicate measurements. (**C**) Secondary structure prediction for selected aptamers.

The aptamer sequences that displayed the highest enrichment levels after nine rounds of selection were subjected to an ELISA-based binding assay to assess their binding capability to human thrombin. To achieve this, human thrombin protein was immobilized on a MaxiSorp plate, then biotinylated aptamers were introduced and incubated with the immobilized protein. Detection of the bound aptamers was accomplished using Streptavidin-HRP and the TMB substrate, with the absorbance measured at 450 nm. Notably, aptamers AYA1809002, AYA1809004, and AYA1809007 exhibited strong binding capabilities, as depicted in Figure 2A. To determine the specificity of the aptamers with robust binding affinities, a competition ELISA-based binding assay was employed (Figure 2B). In this approach, non-biotinylated aptamers in a one-hundred-fold excess relative to their biotinylated counterparts were introduced to the wells containing the respective biotinylated aptamers. If binding is specific, the non-biotinylated aptamers outcompete the biotinylated ones, leading to a significant reduction in absorbance at 450 nm. As demonstrated in Figure 2B, all three tested aptamers were effectively outcompeted by the corresponding non-biotinylated aptamers in a one-hundred-fold excess, confirming their specific binding to the thrombin protein. The predicted secondary structures of the selected aptamers are displayed in Figure 2C. For secondary structure prediction, the RNAfold Webserver, available online, was utilized by adapting it for single-stranded DNA prediction. This prediction technique employs the concept of the Minimum Free Energy (MFE) secondary structure to identify the structure with the lowest free energy. The prediction minimizes the thermodynamic free energy of the nucleic acid molecule to forecast its structure. All the chosen aptamers revealed a hairpin structure characterized by unpaired loop sizes ranging from six to eleven nucleotides and MFE values varying from −7.3 kcal/mol to −9.41 kcal/mol (see Figure 2C). The presence of such a hairpin structure along with unpaired loops in aptamers’ secondary structures is important for their stability, binding affinity, specificity, and adaptability to various targets. The loops, which are unpaired regions, can participate in interactions with the target molecule, thereby contributing to the binding affinity and specificity of the aptamer. The loops can potentially fold into structures that mimic the shape and properties of the binding pocket of the target molecule, which can enhance the aptamer’s ability to bind tightly to its target by fitting into the binding site with complementary interactions. Importantly, it is worth noting that the selected aptamers are prominently rich in G nucleotides, which implies the potential formation of G quadruplex structures. This structural motif has been identified as being common in many DNA aptamers, and might contribute to the enhanced stability of these aptamers [38].

### 3.3. In Vitro Assessment of Aptamers to Evaluate Their Effectiveness in Modulating the Coagulation Cascade

**Figure 3:**
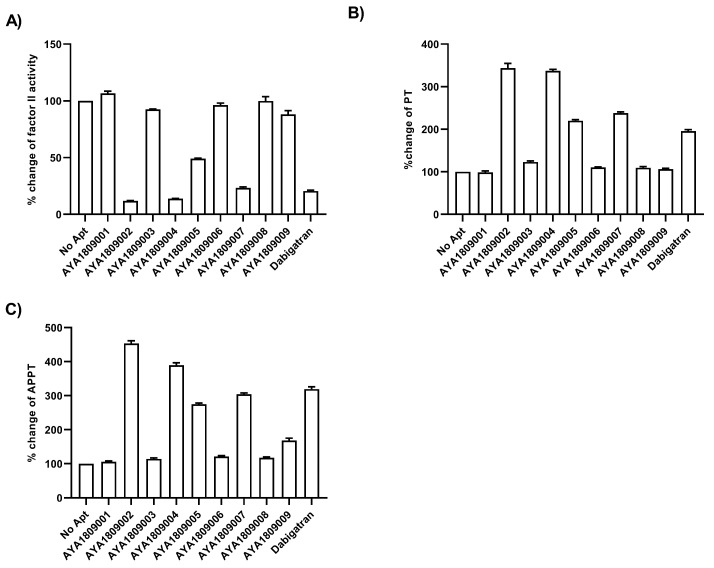
Effect of enriched aptamers on Factor II activity, Prothrombin time (PT), and Activated Partial Thromboplastin Time (APTT). Human plasma was incubated in the absence or presence of 2 µM of either Pradaxa or thrombin aptamers for 2 h at room temperature. (**A**) Inhibition of Factor II Activity by thrombin aptamers as compared to dabigatran was determined by measuring Factor II activity in citrated plasma. Increase of PT time (**B**) and APTT time (**C**) by thrombin aptamers as compared to dabigatran was measured in human citrated plasma. All measurements were conducted using an ACLTOP coagulation analyzer manufactured by Instrumentation Laboratory. The relative activity of Factor II, PT, and APTT time was determined by comparing the treated plasma sample to the untreated one. Each bar shows the average of duplicate measurements.

The nine enriched aptamers underwent testing to evaluate their ability to modulate the coagulation cascade and act as direct thrombin inhibitors, similar to dabigatran. They were tested for their ability to inhibit Factor II activity and extend both prothrombin time (PT) and activated partial thromboplastin time (APTT) (Figure 3). Aptamers AYA1809002, AYA1809004, and AYA1809007 demonstrated superior inhibitory effects on Factor II (Figure 3A) and the most pronounced efficacy in extending both PT (Figure 3B) and APTT (Figure 3C) times. The change of control (untreated condition) served as the baseline, with a normalization of 100%, with the efficacy of the aptamers in altering factor II activity, PT, and APTT presented as a percentage change normalized against the control. At a concentration of 2 µM, AYA1809002 and AYA1809004 exhibit a remarkable reduction in Factor II activity (more than 85%), while AYA1809007 exhibits a decrease in Factor II activity (more than 75%) when compared to Pradaxa (80%). Regarding PT time, AYA1809002 and AYA1809004 demonstrate three-fold increases, while AYA1809007 and dabigatran result in two-fold increases. For APTT, AYA1809002 and AYA1809004 demonstrate an approximate four-fold increase in APTT time, whereas AYA1809007 and dabigatran lead to a three-fold increase. The effects of the aptamers, including the inhibitory effects on Factor II and the extension of PT and APTT time, are comparable to those of dabigatran utilized as a control.

### 3.4. Binding Affinity of Aptamers AYA1809002 and AYA1809004

**Figure 4:**
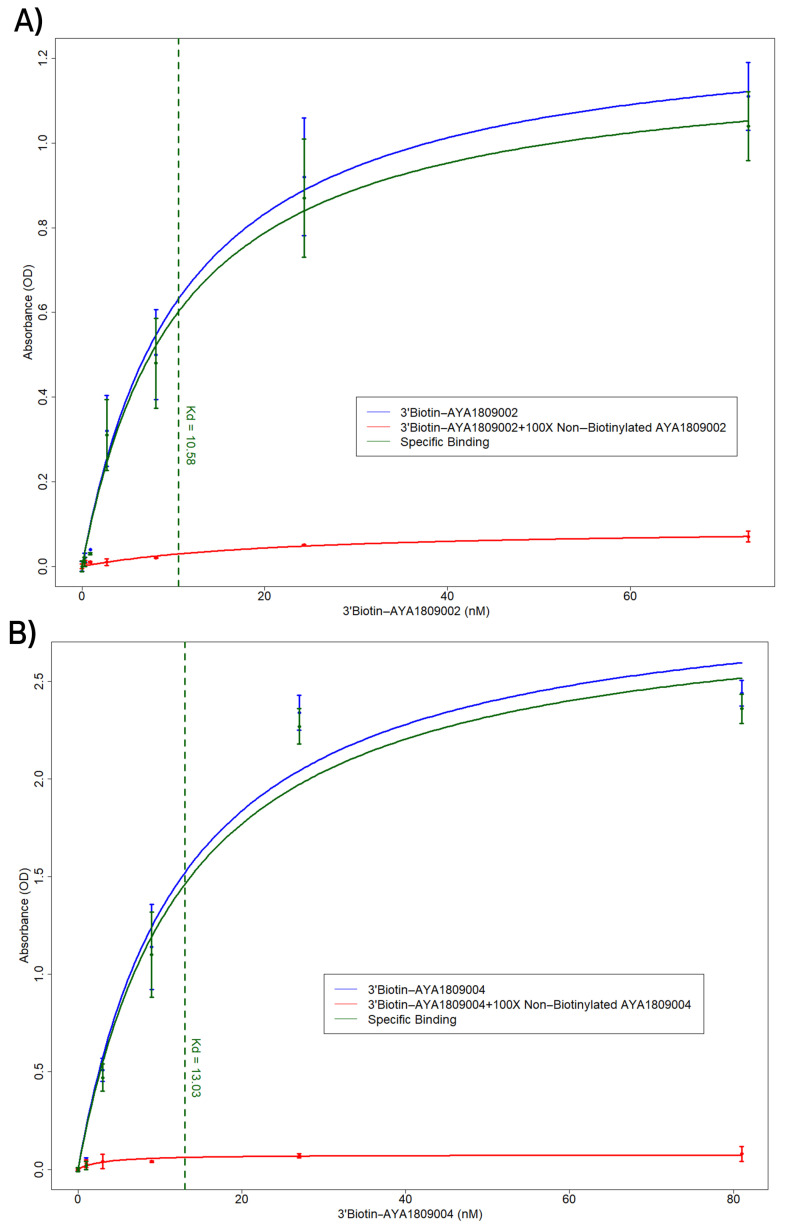
ELISA-based competition assay to determine the affinity constant (Kd) for the aptamers AYA1809002 and AYA1809004. The indicated concentration of either 5${}^{\prime }$ Bioitn-AYA1809002 (**A**) or 5${}^{\prime }$ Bioitn-AYA1809004 (**B**) was incubated with thrombin protein immobilized on a 96-well ELISA plate in the absence or presence of a 100-fold excess of non-biotinylated AYA1809002 or AYA1809004, respectively. Absorbance was measured after incubation with streptavidin horseradish peroxidase bound to the biotinylated aptamer in the presence of the TMB substrate. Each bar shows the average of duplicate measurements. The binding affinity of AYA1809002 to thrombin is estimated to be 10 nM, while that of AYA1809004 is estimated to be 13 nM.

**Figure 5:**
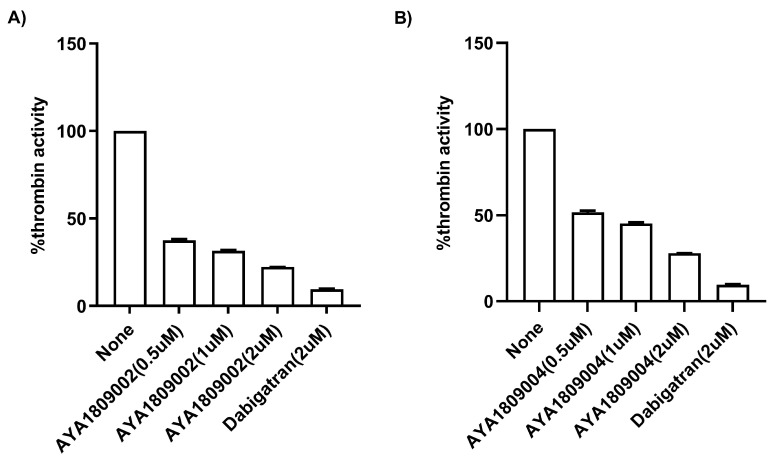
Direct inhibition of thrombin activity by AYA1809002 (**A**) and AYA1809004 (**B**) in a dose-dependent manner. Different concentrations of aptamers, dabigatran at 2 µM, and buffer were added to their respective wells on the test plate. Subsequently, a thrombin enzyme mixture was added to each sample well. Following an incubation period of 15 min, a substrate mixture was introduced into each sample. Fluorescence measurements were taken using a SYNERGY/HTX multi-mode reader (BioTek) with excitation/emission wavelengths set at 360/460 nm. The measurements were recorded for 45 min at a temperature of 37 °C. Each bar shows the average of duplicate measurements.

The combination of binding affinity and in vitro activity data helped to identify the two aptamers AYA1809002 and AYA1809004 as promising candidates for potential therapeutic applications. The binding affinities of the two most potent aptamers, AYA1809002 and AYA1809004, were assessed using an ELISA-based competition assay. The dissociation constant (Kd) was subsequently determined as well (Figure 4). The assay involved incubating the indicated concentrations of either 5${}^{\prime }$ Bioitn-AYA1809002 (Figure 4A) or 5${}^{\prime }$ Biotin-AYA1809004 (Figure 4B) with thrombin protein immobilized on a 96-well ELISA plate. This was done in the absence or presence of a 100-fold excess of non-biotinylated AYA1809002 or AYA1809004, respectively. Absorbance was measured after incubation with streptavidin-horseradish peroxidase bound to the biotinylated aptamer in the presence of TMB substrate. The estimated binding affinity of AYA1809002 to thrombin was 10 nM, while that of AYA1809004 was 13 nM.

### 3.5. Direct Thrombin Activity Inhibition by AYA1809002 and AYA1809004 in a Dose-Dependent Manner

The direct binding and inhibitory capabilities of AYA1809002 and AYA1809004 aptamers on thrombin were evaluated using a Thrombin Inhibitor Screening Assay Kit (fluorometric). The procedure was carried out following the standard protocol provided with the kit. The protocol includes incubating the samples with thrombin followed by the addition of a substrate. The fluorescence intensity is then measured over time to assess the thrombin inhibitory potential of the sample. The kit utilizes the enzymatic activity of thrombin to hydrolyze a synthetic substrate based on AMC (7-amino-4-methylcoumarin), resulting in the liberation of AMC. The released AMC is detectable through fluorescence measurement at an excitation/emission wavelength of 360/460 nm. When thrombin-specific inhibitors are present, the degree of cleavage reaction is diminished or entirely suppressed. As depicted in Figure 5, aptamer AYA1809002 (Figure 5A) and aptamer AYA1809004 (Figure 5B) demonstrated dose-dependent inhibition of thrombin activity. This provides confirmation that the aptamers both bind directly to thrombin and effectively inhibit its enzymatic activity.

### 3.6. Dose-Dependent Effect of AYA1809002 and AYA1809004 on Factor II Activity and PT (Prothrombin Time) in Whole Blood

**Figure 6:**
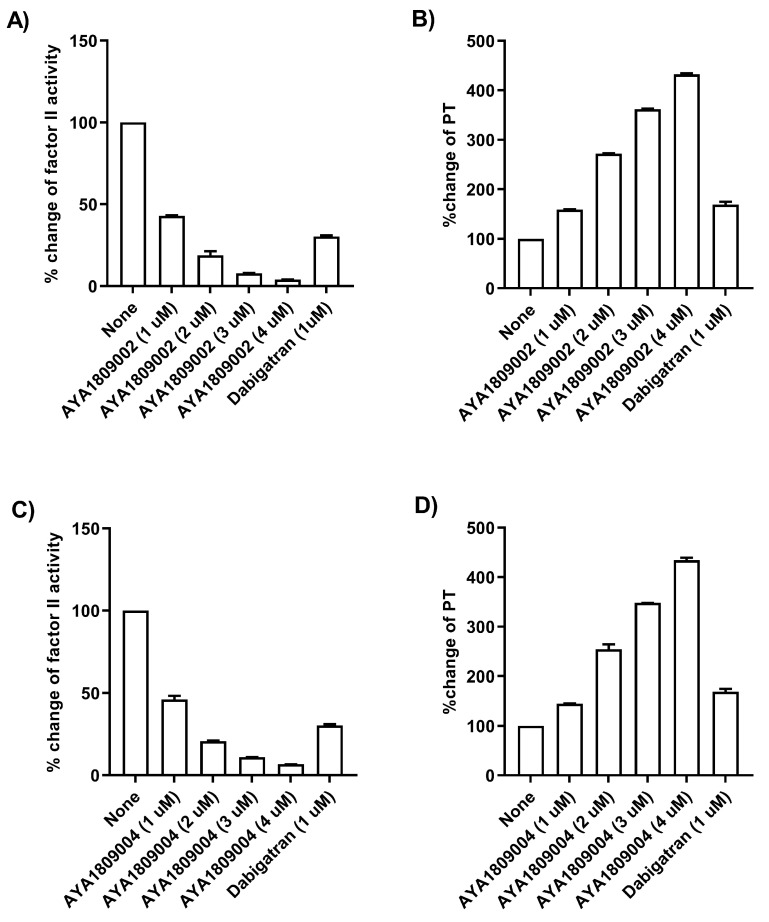
Effect of AYA1809002 and AYA1809004 aptamers on Factor II activity and Prothrombin time (PT) in whole blood in a dose-dependent manner.Whole human blood collected in citrate-treated tubes was incubated in the absence or presence of different concentrations of thrombin aptamers. Dabigatran at a concentration of 1 µM was used as a control. For measurement of Factor II activity and PT time on ACLTOP coagulation analyzer, plasma was separated by centrifugation. Inhibition of Factor II activity by the thrombin aptamers AYA1809002 (**A**) and AYA1809004 (**C**) was determined as compared to non-treated blood. Increase of PT by thrombin aptamers AYA1809002 (**B**) and AYA1809004 (**D**) was measured as compared to non-treated blood. Each bar shows the average of duplicate measurements.

To facilitate future applications, gaining a comprehensive understanding of the activity of aptamers in whole blood is of paramount importance. To assess their effectiveness, we tested the selected aptamers at various concentrations in whole blood to evaluate their impact on Factor II activity and PT time. Human blood samples were collected in citrate-treated tubes. The blood samples were then incubated at room temperature for 2 h in the absence or presence of varying concentrations of thrombin aptamers. To measure Factor II activity and PT time on the ACLTOP coagulation analyzer, plasma was obtained by centrifuging the samples at 1500× *g* for 10 min at 4 °C. For the dose dependence study, concentrations of 1 µM, 2 µM, 3 µM, and 4 µM of AYA1809002 and AYA1809004 were utilized. Figure 6 illustrates a clear dose-dependent decrease in Factor II activity for both aptamers (Figure 6A,C). Additionally, as the concentration of the aptamers increases there is a corresponding increase in prothrombin time (Figure 6B,D). Dabigatran at a concentration of 1 µM was used as a control. These results demonstrate a dose-dependent effect of AYA1809002 and AYA1809004 on both Factor II activity and PT time, suggesting that the selected aptamers impact the overall coagulation process.

### 3.7. Stability of Selected Aptamers in Whole Blood

**Figure 7:**
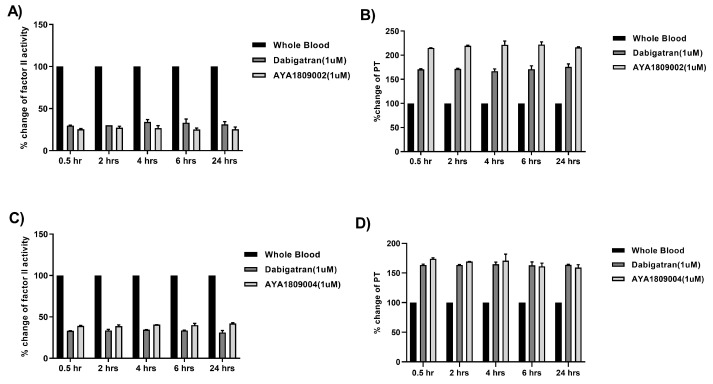
Stability of the selected aptamers in whole blood at room temperature. Citrated blood collected from a donor was incubated in the absence or presence of 1 µM of AYA1809002, AYA1809004, or Dabigatran for the indicated time. Factor II activity (**A**,**C**) and PT time (**B**,**D**) were measured for each sample. The relative activity was determined by dividing the measured activity of the treated whole blood sample by the activity of the corresponding untreated sample collected at the same time point. Each bar shows the average of duplicate measurements.

The stability of aptamers in whole blood holds significant importance for their future applications, as it provides insights into their longevity and potential as anticoagulants or thrombin inhibitors compared to the reference drug dabigatran. To evaluate the stability of the selected aptamers AYA1809002 and AYA1809004 and compare them to dabigatran, citrated blood collected from a donor was incubated at room temperature in the absence or presence of 1 µM of AYA1809002, AYA1809004, or dabigatran for 0.5, 2, 4, 6, and 24 h. At each time point, blood samples from both the treated and untreated groups were collected and plasma was obtained through centrifugation. As depicted in Figure 7, AYA1809002 and AYA1809004 demonstrated stability and effectiveness for up to 24 h. This finding suggests that the aptamers retained their inhibitory properties against thrombin during the entire 24 h period of incubation. By performing the same experiment using whole blood at 37 °C for 24 h, we aimed to evaluate the stability and effectiveness of the aptamers in vitro under physiological conditions, specifically at body temperature (Supplemental Figure S1). The fact that the effectiveness of the aptamers remained intact for the entire 24 h duration at 37 °C suggests that the aptamers are stable in vitro and capable of exerting their anticoagulant effects even under conditions that mimic the human body’s physiological temperature.

To further substantiate the stability of the aptamers in an in vitro environment, we replicated the stability assay using whole blood at 37 °C. Across different time points, we employed biotinylated reverse complement sequences in conjunction with streptavidin-coated magnetic beads to carefully extract the aptamers from the blood samples. Upon elution from the magnetic beads, the aptamers were subjected to analysis using a fragment analyzer. The outcomes of this investigation are depicted in Supplemental Figure S3. The traces of the aptamers at various time intervals confirm that the aptamers remain structurally intact even after 24 h of incubation in citrated blood at 37 °C. Recognizing that this study represents a preliminary phase in assessing the stability of these aptamers in an in vivo context, in order to enhance the durability of the aptamers within the bloodstream we integrated the well-established stabilizing agents PEG, TEG, and cholesterol into our aptamer designs (see Section 3.10). In summary, our methodology encompassed a comprehensive assessment of aptamer stability in vitro, combining coagulation cascade modulation with analysis of their structural integrity in whole citrated blood at physiologically relevant temperatures.

### 3.8. Restoration of Factor II Activity and PT Time with Reverse Complement to AYA1809002 in Whole Blood

**Figure 8:**
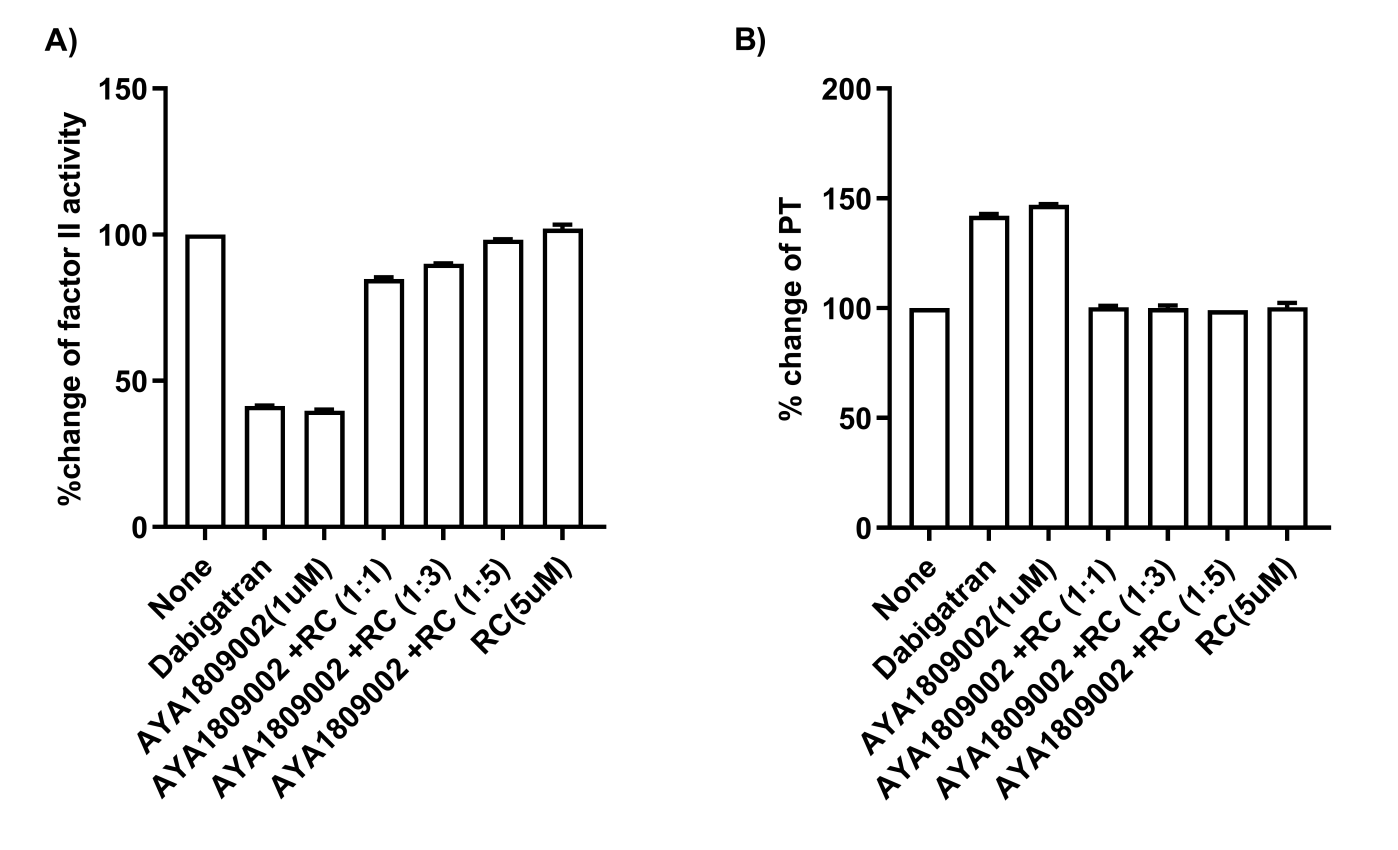
Restoration of Factor II Activity and PT Time using the reverse complement to AYA1809002 in whole blood. Citrated blood collected from a donor was incubated in the absence or presence of 1 µM AYA1809002 or dabigatran at room temperature. After 2 h, the indicated concentration of the reverse complement strand was added to the citrated blood sample. After an additional incubation period, plasma was collected per the existing protocol and Factor II activity (**A**) and PT time (**B**) were measured. Each bar shows the average of duplicate measurements.

The ability to reverse or counteract the anticoagulant effects of thrombin inhibitors is essential in situations where immediate hemostasis is required, such as during emergency surgeries, major bleeding events, or in patients who experience complications associated with anticoagulant therapy. One potential approach to reverse the anticoagulant effect of aptamers is to use their reverse complement sequences. The reverse complement sequence is designed to bind to and neutralize the aptamer’s activity. To investigate the ability of the reverse complement strand to reverse the anticoagulant effect of AYA1809002, citrated blood collected from a donor was incubated in the absence or presence of 1 µM AYA1809002 at room temperature. After 2 h, the indicated concentration of the reverse complement strand was added to the citrated blood sample with the aptamer. After an additional incubation period, plasma was collected per the existing protocol and Factor II activity (Figure 8A) and PT time (Figure 8B) were measured. Based on Figure 8, it is evident that the reverse complements of the aptamers completely inhibit their activity and effectively restore both Factor II activity and PT time even at a 1:1 ratio with the aptamers. The reverse complements act as antagonists to the aptamers, effectively reversing their impact on the coagulation cascade.

### 3.9. Clot-Bound Thrombin

**Figure 9:**
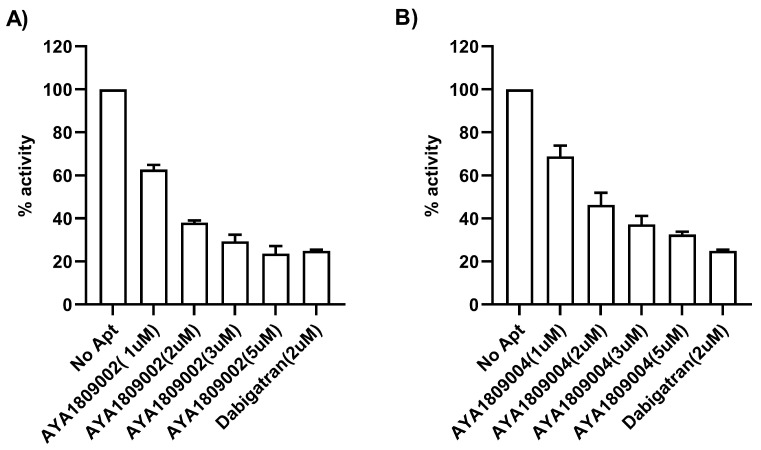
Inhibition of clot-bound thrombin by the selected aptamers AYA1809002 (**A**) and AYA1809004 (**B**). Fibrin clots were formed from platelet-rich plasma. Different concentrations of aptamers, Dabigatran at 2 µM, and buffer were added to the respective wells containing the washed clots on the test plate. Following a 15 min incubation period, a substrate mixture was introduced into each sample. Fluorescence measurements were taken using a SYNERGY/HTX multi-mode reader (BioTek) with the excitation/emission wavelengths set at 360/460 nm. The measurements were recorded for 90 min at a temperature of 37 °C. Each bar shows the average of duplicate measurements.

Clot-bound thrombin refers to thrombin that becomes localized and trapped within a blood clot. In this state, it continues to promote fibrin formation, resulting in the further growth and stabilization of the clot. To ensure comprehensive evaluation of the selected aptamers, it is essential to test their inhibitory potential against thrombin when it is in the clot-bound stage. For the inhibition study of clot-bound thrombin, we utilized the method previously described in [32] with a few modifications. Clots were generated in human platelet-rich plasma by adding CaCl${}_{2}$ and incubating for 2 h at 37 °C. After washing the clots, they were transferred to TBS buffer containing either AYA1809002, AYA1809004, or dabigatran. Following a brief incubation, thrombin fluorogenic substrate was added to each sample and fluorescence measurements were conducted. Using this approach, we employed the ability of clot-bound thrombin to cleave a synthetic AMC-based substrate to release AMC. Based on the data presented in Figure 9, both aptamers demonstrated the ability to inhibit clot-bound thrombin in a concentration-dependent manner, with aptamer AYA1809002 exhibiting a higher level of inhibition compared to AYA1809004. Notably, the inhibition of clot-bound thrombin with the two aptamers is comparable to that of dabigatran.

### 3.10. Modified AYA1809002 and AYA1809004 Aptamers

To enhance their stability in the bloodstream, the selected aptamers were chemically modified through crosslinking with TEG, PEG, and cholesterol moieties. The modified AYA1809002 and AYA1809004 aptamers were then evaluated to determine their impact on Factor II activity, PT, and APTT in plasma. Additionally, the potential reversal of their effects was investigated using reverse complement sequences that correspond to each aptamer (Supplemental Figures S2 and S3). Figure S2 clearly illustrates that the modified AYA1809002 exhibits a similar impact on Factor II, PT, and APTT compared to the unmodified aptamers. Notably, when the reverse complement is applied it effectively reverses the observed effects. The modified AYA1809004 aptamer is less effective compared to the non-modified aptamer (Supplemental Figure S3). The cholesterol-modified AYA1809004 showed no significant impact on Factor II activity, PT, or APTT. However, the PEG- and TEG-modified variants demonstrated noticeable inhibition of Factor II activity and a slight increase in PT and APTT times. The reverse complement of AYA1809004 effectively restored the impact on Factor II activity, PT, and APTT for all modified aptamers, suggesting that the reverse complement sequence can counteract the inhibitory effects observed with the modified aptamer.

### 3.11. Safety of the AYA1809002 and AYA1809004 Aptamers

Aptamers are generally considered to have a good safety profile thanks to their biocompatibility and similarity to naturally occurring nucleic acids. The evaluation of aptamer safety involves assessing their stability, specificity, immunogenicity, and mutagenicity.

#### 3.11.1. Immunogenicity Assessment of AYA1809002 and AYA1809004 Aptamers

**Figure 10:**
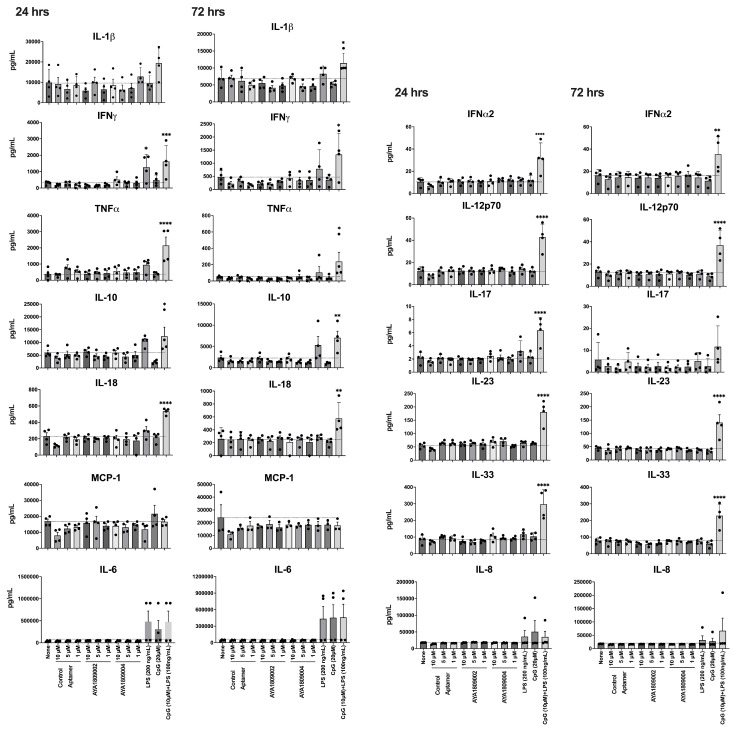
Immunogenic response of AYA1809002 and AYA1809004. Human PBMCs were stim- ulated with or without AYA1809002, AYA1809004, control aptamer, LPS, ODN 1826, and LPS+ODN 1826 at 37 °C for 24 and 72 h. The amount of cytokines secreted from the hPBMCs was assessed using a LEGENDplex Human Inflammation Kit. Soluble analytes were quantified using flow cytometry and analyzed with BioLegend’s LEGENDplex${}^{\mathrm{TM}}$ software. Each dot represents an individual donor (n = 4). Data are graphed as the mean value ± SEM. The *p* values were determined with one-way ANOVA, Dunnett’s multiple comparison test. ${}^{*}$ denotes *p*≤ 0.05, ${}^{**}$ denotes *p*≤ 0.01, ${}^{***}$ denotes *p*≤ 0.001. ${}^{****}$ denotes *p*≤ 0.0001.

To investigate the potential immunogenicity of AYA1809002 and AYA1809004 using an in vitro human model, we measured the levels of human inflammatory cytokines and chemokines induced by varying doses of AYA1809002 and AYA1809004 in cultured human peripheral blood mononuclear cells (PBMCs). The assessed cytokines included IL-1β, IFN-α2, IFN-γ, TNF-α, MCP-1 (CCL2), IL-6, IL-8 (CXCL8), IL-10, IL-12p70, IL-17A, IL-18, IL-23, and IL-33. The PBMCs were subjected to the indicated conditions, and the culture media were collected at 24 and 72 h post-treatment, as illustrated in Figure 10. For human PBMCs treated with 1, 5, or 10 µM concentrations of AYA1809002 and AYA1809004, the secretion of IL-1β, IFN-α2, IFN-γ, TNF-α, MCP-1 (CCL2), IL-6, IL-8 (CXCL8), IL-10, IL-12p70, IL-17A, IL-18, IL-23, and IL-3 was comparable to that of cells treated with mock or control aptamers at both 24 and 72 h. The positive control groups, especially a combination of LPS (100 ng/mL) and ODN 1826 (10 µM), exhibited elevated production of IL-1β, IFN-α2, IFN-γ, TNF-α, IL-6, IL-8 (CXCL8), IL-10, IL-12p70, IL-17A, IL-18, IL-23, and IL-3 as compared to mock-treated cells at both 24 and 72 h following stimulation at the specified doses. Collectively, these findings suggest that AYA1809002 and AYA1809004 do not exhibit immune-stimulating properties. These observations are in agreement with the study by Thiel et al. [39], which demonstrated that a smooth muscle cell-targeted RNA aptamer did not induce increased release of the inflammatory cytokines IL-6, IFN-β, or IFN-γ from human PBMCs. Based on our investigations, AYA1809002 and AYA1809004 seem to possess a favorable safety profile.

#### 3.11.2. Mutagenicity Assessment of AYA1809002 and AYA1809004 Aptamers Using the Ames Test

**Figure 11:**
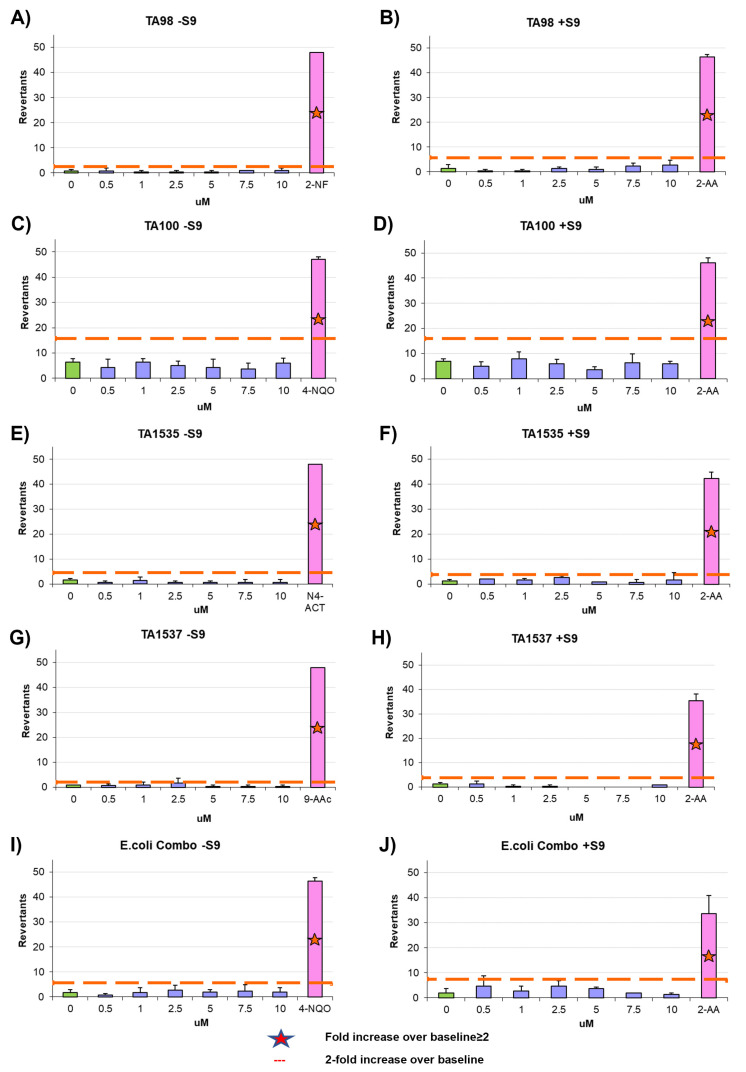
Microbial mutagenicity test for AYA1809002 (**A**–**J**). Assessment of the aptamer’s mutagenic potential involved subjecting *S. typhimurium* strains (TA98, TA100, TA1535, TA1537) and *E. coli* strains (wp2[pkM101] and wp2 uvrA) to varied concentrations of the aptamer. Positive and negative controls were included. The aptamer AYA1809002 was applied at concentrations ranging from 0.5 µM to 10 µM. This assay was conducted both in the presence and absence of metabolic activation, which was facilitated by liver homogenate S9. Results were obtained from three independent experiments, and the data are presented as the mean ± standard deviation (SD).

**Figure 12:**
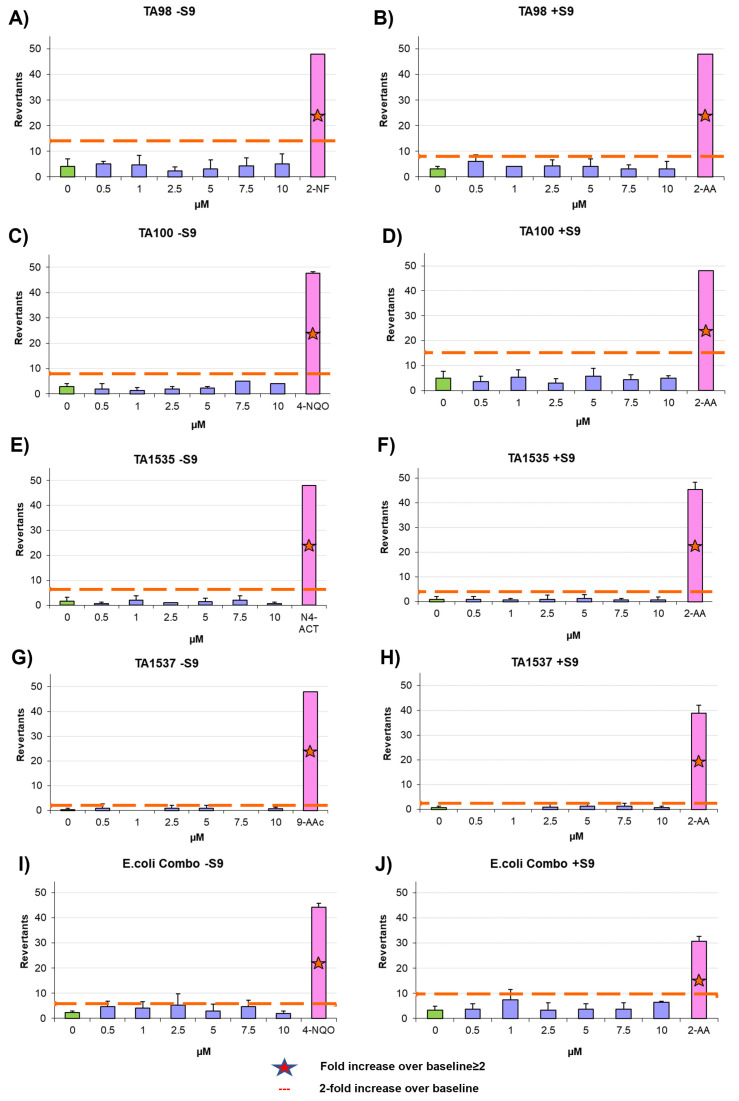
Microbial mutagenicity test for AYA1809004 (**A**–**J**). Assessment of the aptamer’s mutagenic potential involved subjecting *S. typhimurium* strains (TA98, TA100, TA1535, TA1537) and *E. coli* strains (wp2[pkM101] and wp2 uvrA) to varied concentrations of the aptamer. Positive and negative controls were included. The aptamer AYA1809004 was applied at concentrations ranging from 0.5 µM to 10 µM. This assay was conducted in both the presence and absence of metabolic activation, which was facilitated by liver homogenate S9. Results were obtained from three independent experiments, and the data are presented as the mean ± standard deviation (SD).

To assess the mutagenicity of the aptamers, we performed Ames test, a widely used assay for evaluating the mutagenicity of chemical compounds. The Ames test is a bacterial reverse mutation assay that detects the ability of a substance to induce mutations in the DNA of certain strains of bacteria. It utilizes various bacterial strains, including *Salmonella typhimurium* strains TA98, TA100, TA1535, and TA1537, as well as the *Escherichia coli* strains wp2 [pKM101] and wp2 uvrA [40,41,42,43]. To evaluate the mutagenic potential of an aptamer, amino acid-requiring bacterial organisms are exposed to different concentrations of the aptamer and mutagenic events are assessed by selecting for reversion events. In this context, the aptamers AYA1809002 and AYA1809004 were supplemented at increasing concentrations ranging from 0.5 µM to 10 µM. Our observations revealed that all tested strains treated with various concentrations of the aptamers exhibited fewer revertants compared to the established baseline represented by the positive control cut-off (see Figure 11 and Figure 12). Taken together, these findings collectively indicate that the aptamers AYA1809002 and AYA1809004 do not exhibit mutagenic properties.

## 4. Discussion

Thrombin plays a crucial role as a central orchestrator in the blood coagulation cascade, encompassing various essential functions such as pro-coagulation, anticoagulation, platelet aggregation, and inflammatory activities. From hirudin, to parenteral direct thrombin inhibitors (lepirudin, desirudin, argatroban, and bivalirudin), to oral direct thrombin inhibitors (ximelagatran and dabigatran), scientists have been working hard to investigate and develop a range of thrombin inhibitors in recent decades. However, the use of thrombin inhibitors is limited or contradicted in clinics due to drug interactions, side effects, risk of bleeding, and cost. The biggest challenge in developing thrombin inhibitors is the need to reduce bleeding-related risks while maintaining high anti-thrombin efficacy. Therefore, developing a thrombin inhibitor that is efficient, safe, affordable, and has minimal side effects remains highly necessary. In this study, we employed theSystematic Evolution of Ligands by Exponential Enrichment (SELEX) method to generate a diverse range of aptamers targeting the active site of thrombin. Notably, during the SELEX procedure we introduced dabigatran as a competitor to enhance the selection specificity.

Employing this approach, we successfully obtained a pool of aptamers that displayed high affinity and selectivity toward thrombin. Within this enriched pool, aptamers AYA1809002 and AYA1809004 demonstrated the highest binding affinity for thrombin among all of the identified candidates. The effectiveness of these aptamers was evaluated in vitro using an FDA-approved ACLTOP instrument in both plasma and whole blood samples. The assessment focused on measuring Factor II activity, PT time (Prothrombin Time), and APTT time (Activated Partial Thromboplastin Time). The results of the study demonstrated a clear dose-dependent effect of both aptamers on Factor II activity. As the concentration of the aptamers increased, there was a corresponding reduction in Factor II activity. This indicates that the aptamers effectively inhibited the functionality of Factor II, a key component of the coagulation cascade. Furthermore, the study revealed an increase in both PT time and APTT time with increasing concentrations of the aptamers. PT time is a measure of the extrinsic pathway of the coagulation cascade, while APTT time assesses the intrinsic pathway [44,45,46,47,48]. These prolonged PT and APTT times indicate that the aptamers impacted the coagulation process and led to a delay in clot formation. These results support the potential utility of the selected aptamers as anticoagulant agents or tools for studying the coagulation process.

Extensive research has been conducted over the years to find reversal agents for anticoagulation therapy. Numerous studies have focused on identifying and evaluating effective strategies to reverse the anticoagulant effects of various anticoagulants, including thrombin inhibitors [49,50,51,52]. The unique properties of aptamers allow for the rational design of antidotes, providing a means to extend the heparin–protamine paradigm to a new class of direct-acting specific anticoagulants [13]. This drug–antidote design technology offers the possibility of novel antidote-based control of antithrombotic activity. As a result, several studies have focused on the development of anticoagulation therapies by selecting DNA aptamers that specifically bind to thrombin, as mentioned earlier. The ability of the reverse complements of the AYA1809002 and AYA1809004 aptamer sequences to restore Factor II activity and PT time provides valuable insights into the regulatory mechanisms involved in the coagulation process. In addition, it highlights the potential of developing therapeutic strategies utilizing reverse complements to counteract the effects of aptamers when necessary. The findings presented in Figure 8 indicate the potential of reverse complements to modulate and fine-tune the coagulation processes by counteracting the effects of aptamers.

Chemical modifications such as crosslinking with TEG, PEG, or cholesterol entities were employed to enhance the stability of the aptamers in the bloodstream. Remarkably, these modifications did not compromise the effectiveness of the aptamers in terms of inhibiting Factor II activity and prolonging PT and APTT times. The modified aptamers retained their inhibitory effects on the measured coagulation parameters, demonstrating their potential as stable and functional candidates for anticoagulation therapy. Aptamers AYA1809002 and AYA1809004 were shown to be safe, as they demonstrated a non-immunogenic nature. This indicates a favorable safety profile for these molecules, making them potentially well-tolerated in therapeutic applications. Additionally, a mutagenicity assessment, specifically the Ames test, confirmed that these aptamers do not possess mutagenic properties. These findings provide strong evidence supporting the safety and biocompatibility of AYA1809002 and AYA1809004, reinforcing their potential as promising candidates for therapeutic interventions. It is worth noting that the selected aptamers are rich in G. This observation suggests the potential formation of G quadruplex structures, a prevalent motif found in numerous DNA aptamers. This structural characteristic could potentially enhance the stability of these aptamers. When Bock and Toole discovered the first single-strand DNA aptamer against human protease thrombin, they discovered that the sequence of GGTTGG was the most conserved sequenced in almost all of their selected clones [53]. Greatly delayed thrombin-catalyzed conversion of fibrinogen to fibrin has been observed at 15 mer with sequences 5${}^{\prime }$-GGTTGGTGTGGTTGG-3${}^{\prime }$. Using this aptamer, Li et al. demonstrated its efficiency to inhibit clot-bound thrombin activity and reduce arterial platelet thrombus formation [54]. Among the enriched aptamers, with the exception of AYA1809001, there were similarities in the sequence motif GGTTGG. AYA1809002 possessed the extended sequence GGTTGGGAGGTTGG, while AYA1809004 had the longer sequence GGTTGGGGGGGTTGG. These sequences are G-rich and have the potential to form G-quadruplex structures, similar to the aptamers studied by Bock and Toole. The selected aptamers exhibit a predicted hairpin structure with unpaired loop sizes ranging from six to eleven nucleotides. The presence of such a hairpin structure, along with unpaired loops in the aptamers’ secondary structures, is important for their stability, binding affinity, specificity, and adaptability to various targets. Hairpin structures can provide conformational flexibility, allowing the aptamer to adapt its structure in order to more optimally interact with the target. This adaptability is crucial for effective binding, as the target molecule might have different conformations or binding requirements. The fact that multiple aptamers share a similar structural feature (a hairpin with unpaired loops) suggests that there might be a common binding mechanism or principle that underlies their interactions with their respective targets.

The selected aptamers were subjected to testing to evaluate their ability to inhibit both fluid-phase thrombin and clot-bound thrombin. This comprehensive assessment allowed us to determine their efficacy in targeting thrombin in different contexts. By inhibiting fluid-phase thrombin, the selected aptamers can directly prevent excessive thrombin activity in the blood, while their ability to inhibit clot-bound thrombin offers the potential to prevent further thrombin-mediated clotting events and inhibit the propagation of the clot. This dual inhibitory action further highlights their versatility and therapeutic value in managing thrombotic conditions.

In this study, we have demonstrated the properties and anticoagulant activity of two anti-thrombin aptamers. Our findings suggest that AYA1809002 and AYA1809004 can be effective, safe, and affordable potential therapies for thrombosis.

## 5. Conclusions

Our primary goal in developing these new anti-thrombin DNA aptamers is to broaden the selection of aptamer candidates that could eventually be considered for clinical applications. Our comprehensive investigation has revealed compelling evidence supporting the efficacy and safety of AYA1809002 and AYA1809004 as potent anti-thrombin candidates. These aptamers demonstrate remarkable performance in vitro in inhibiting thrombosis, effectively countering excessive clot formation, and their activities are easily reversible by administration of the antidote. Furthermore, a comprehensive assessment of their safety profile through in vitro studies demonstrated minimal negative impacts, reinforcing their potential in clinical applications. It is important to note that in vitro evaluations serve as only an initial step in assessing the effectiveness of aptamers and their potential clinical applications. Further studies, including in vivo investigations and clinical trials, remain necessary to validate these findings and determine the safety and efficacy of the aptamers in more realistic and dynamic physiological environments.

## Figures and Tables

**Table 1:** The top nine most enriched sequences after nine rounds of selection.

Name	Sequence
AYA1809001	GGTAGCGTAAGGATGCGCAAGTTTAATTGCCATATGCCAT
AYA1809002	GCGGTGTTGCTGGGGTTGGGAGGTTGGAGGAAGCAATCGC
AYA1809003	GTGTAGGATGGGTGGGTGGGTCACATTTAAGATATCCTGG
AYA1809004	TCGTCAAGGTACGGTTGGGGGGGTGGGTGTTGACGTTGAA
AYA1809005	GCGGATGGATGGTGAGGTTGGGAGCTTTCATTGGAACTAA
AYA1809006	GGGTAGGGTGGTGAGATGAAATCTCTAGGGTGATCGTTCT
AYA1809007	AGGGGCTCTAGGGTGGGTAGGATGGTGAGACCGCAGCGT
AYA1809008	CGTGGGGATGGGTGGGTGGAGGAGCGATGATGGCCTACGA
AYA1809009	GGTAGCGTAAGGATGCGCAAGTTTAATTGCCATATGCCAT

## Data Availability

This article has accompanying Supplementary Files. All data generated or analyzed during this study are included in the published article and its Supplementary Files.

## Supplementary Materials

The following supporting information can be downloaded at: https://www.mdpi.com/article/10.3390/cells12182230/s1, Figure S1: Stability of selected aptamers in whole blood at 37 °C. Figure S2: Effect of modified AYA1809002 aptamer on Factor II activity, PT, and APTT and reversal potential using Reverse Complement to AYA1809002. Figure S3: Effect of modified AYA1809004 aptamer on Factor II activity, PT, and APTT and reversal potential using Reverse Complement to AYA1809004. Figure S4: Stability analysis of aptamers after 2-, 6- and 24-hours incubation in citrated blood at 37 °C. Table S1: Modified and Control Aptamer Sequences Used in the Study.
