# Supplementary material for: New High-Affinity Thrombin Aptamers for Advancing Coagulation Therapy: Balancing Thrombin Inhibition for Clot Prevention and Effective Bleeding Management with Antidote
Source: Cells. 2023 Sep 7;12(18):2230. doi: 10.3390/cells12182230 (PMC10526462; doi:10.3390/cells12182230)
Supplement: Supplementary file 1 [file cells-12-02230-s001.zip › cells-2545052-supplementary.pdf]

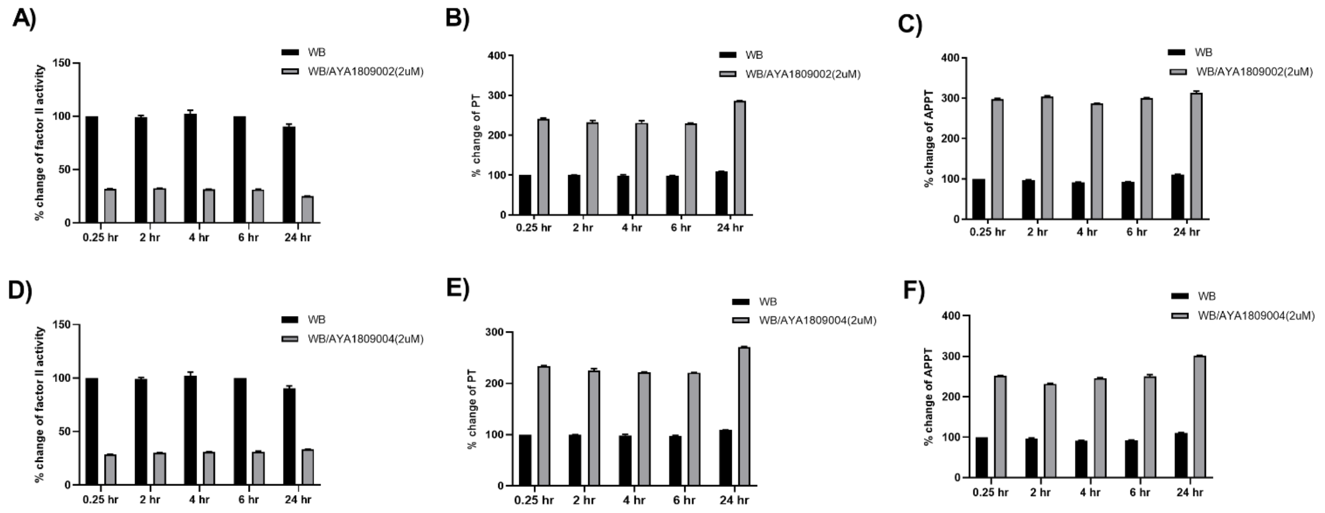

Supplemental Figure S1. Stability of selected aptamers in whole blood at 37^0 C.
Stability experiments were performed using whole blood at 37^0 C. Citrated blood collected from a donor was incubated in the absence or presence of 1 uM AYA1809002 or AYA1809004 at 37^0C. Factor II activity **(A** and **D)**, PT time **(B** and **E)** and APTT time **(C** and **F)** were measured for each sample. The relative activity was determined by dividing the measured activity of the treated whole blood sample by the activity of the corresponding untreated sample collected at the same time point. Each bar is an average of duplicate measurements.

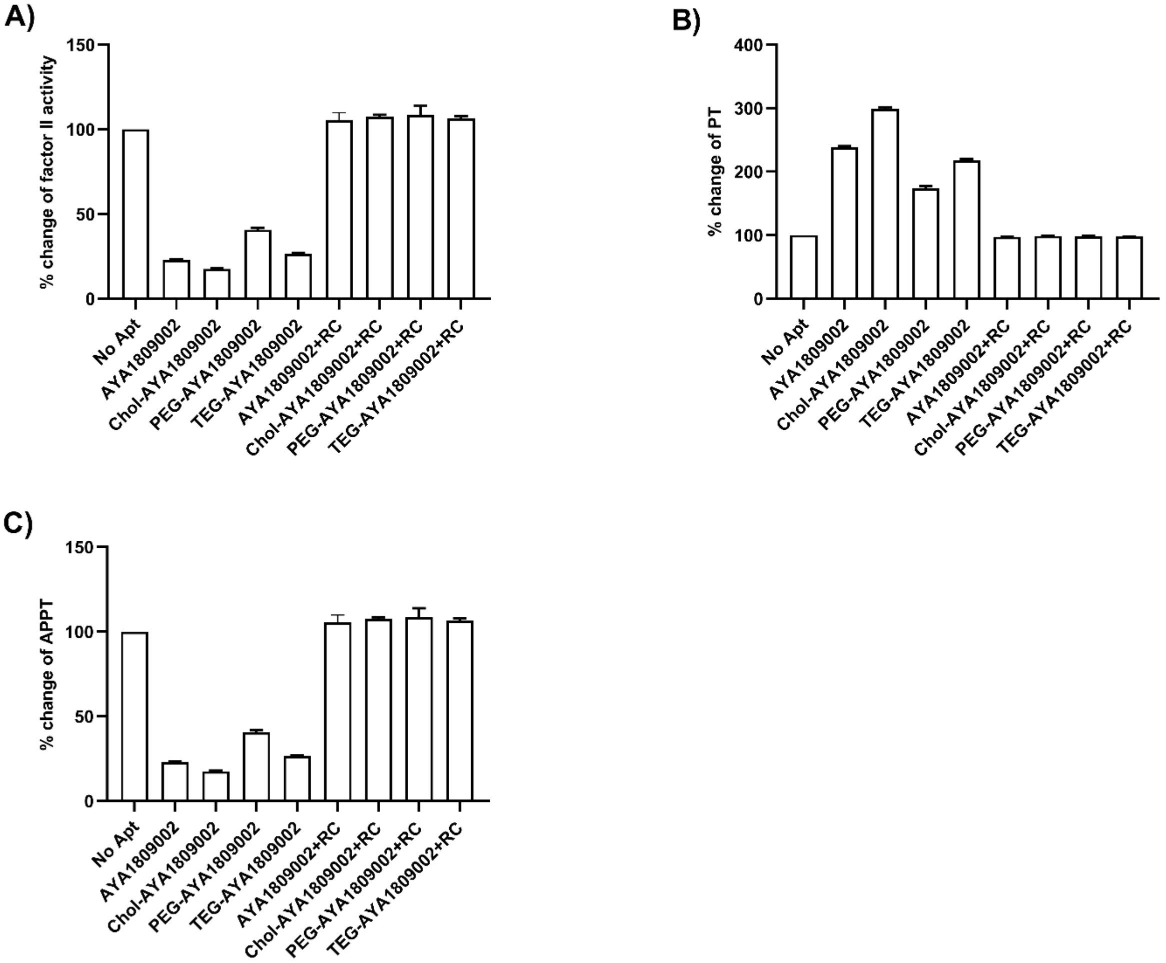

Supplemental Figure S2. Effect of modified AYA1809002 aptamer on Factor II activity, PT, and APTT and reversal potential using Reverse Complement to AYA1809002.

Human citrated plasma was incubated in the absence or presence of 2 uM of unmodified AYA1809002 or Cholesterol-, PEG-or TEG-modified AYA1809002 aptamers for 1 hour at room temperature. The indicated samples were supplemented with 5 µM of the reverse complement strand to AYA1809002. After an additional 2-hour incubation, Factor II activity **(A)** and PT time **(B)** and APTT time **(C)** were measured. The relative activity of Factor II, PT and APTT time was determined by comparing the treated plasma sample to the untreated one. Each bar is an average of duplicate measurements.

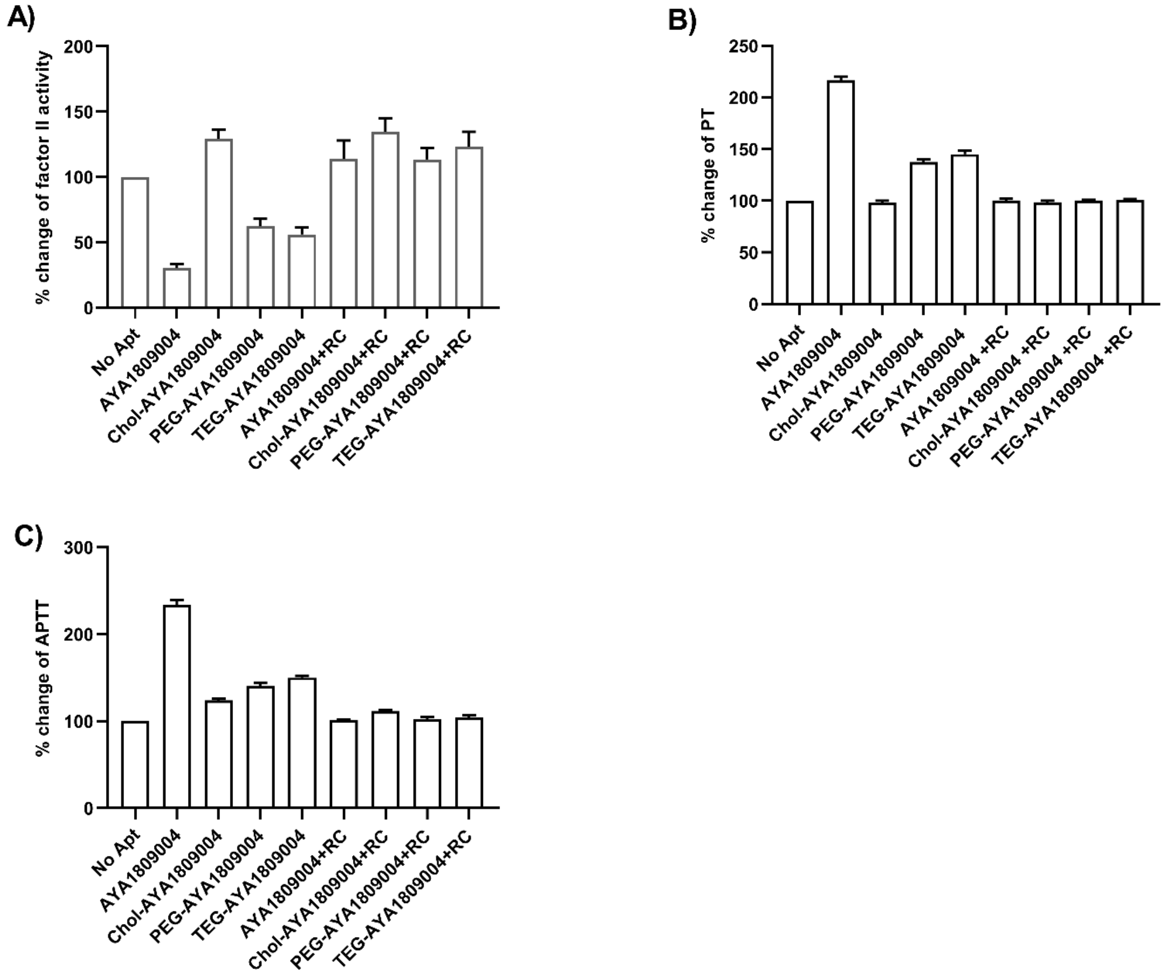

Supplemental Figure S3. Effect of modified AYA1809004 aptamer on Factor II activity, PT, and APTT and reversal potential using Reverse Complement to AYA1809004.

Human citrated plasma was incubated in the absence or presence of 2 uM of unmodified AYA1809004 or Cholesterol-, PEG-or TEG-modified AYA1809004 aptamers for 1 hour at room temperature. The indicated samples were supplemented with 5 µM of the reverse complement strand to AYA1809004. After an additional 2-hour incubation, Factor II activity **(A)** and PT time **(B)** and APTT time **(C)** were measured. The relative activity of Factor II, PT and APTT time was determined by comparing the treated plasma sample to the untreated one. Each bar is an average duplicate measurements.

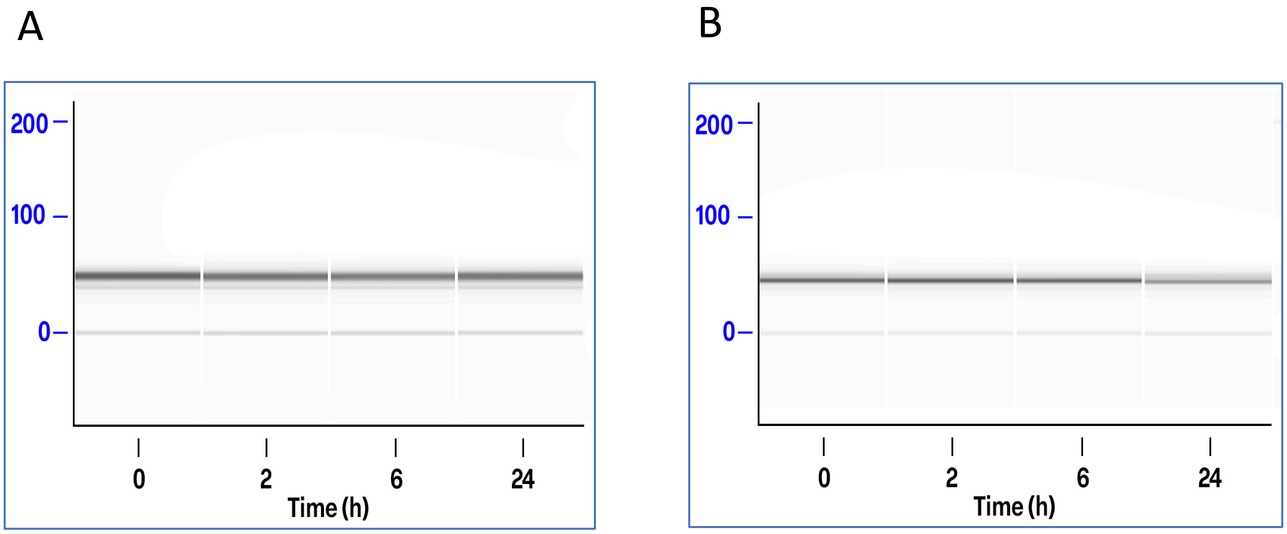

Supplemental Figure S4. Stability analysis of aptamers after 2-, 6- and 24-hours incubation in citrated blood at $37^0\mathrm{C}$. Analysis was performed using Fragment analyzer (Agilent Technologies). A) aptamer AYA1809002; B)aptamer AYA1809004.

Modified Aptamers	Sequences
3'PEG_AYA1809002	GCGGTGTTGCTGGGGTTGGGAGGTTGGAGGAAGCAATCGC/3Sp18/
3'TEG_AYA1809002	GCGGTGTTGCTGGGGTTGGGAGGTTGGAGGAAGCAATCGC/3Sp9/
3'Chol_AYA1809002	GCGGTGTTGCTGGGGTTGGGAGGTTGGAGGAAGCAATCGC/3CholTEG/
3'PEG_AYA1809004	TCGTCAAGGTACGGTTGGGGGGGTGGGTGTTGACGTTGAA/3Sp18/
3'TEG_AYA1809004	TCGTCAAGGTACGGTTGGGGGGGTGGGTGTTGACGTTGAA/3Sp9/
3'Chol_AYA1809004	TCGTCAAGGTACGGTTGGGGGGGTGGGTGTTGACGTTGAA/3CholTEG/
Control aptamer	GCGCGGTCCCGATTTGGTGTAAAATTCCCTCAGCCCTACA

Table S1. Modified and Control Aptamer Sequences Used in the Study.
